# Contemporary outcomes of childhood aortic coarctation interventions: a national registry analysis of mortality, reinterventions and hospital resource use

**DOI:** 10.1136/heartjnl-2024-325346

**Published:** 2025-07-08

**Authors:** Dan Mihai Dorobantu, Qi Huang, Ferran Espuny-Pujol, Kate L Brown, Rodney Franklin, Sonya Crowe, Christina Pagel, Serban Stoica

**Affiliations:** 1 Cardiology Department, Bristol Heart Institute, Bristol, UK; 2 Children's Health and Exercise Research Centre (CHERC), University of Exeter Medical School, Exeter, UK; 3 Clinical Operational Research Unit, Department of Mathematics, University College London, London, UK; 4 Heart and Lung Division, Great Ormond Street Hospital NIHR Biomedical Research Centre, London, UK; 5 Department of Paediatric Cardiology, Royal Brompton and Harefield National Health Service Foundation Trust, London, UK; 6 University of Bristol Medical School, Bristol, UK; 7 Paediatric Cardiac Surgery Department, Bristol Royal Hospital for Children, Bristol, UK

**Keywords:** Aortic Coarctation, Congenital heart disease, Heart Septal Defects, Ventricular

## Abstract

**Background:**

Coarctation of the aorta (CoA) has good modern results, but large multicentre longitudinal data on outcomes, especially hospital resource utilisation through childhood and adolescence, are not available.

**Methods:**

All patients with CoA treated between 2000 and 2017 in England and Wales were linked to hospital and outpatient records through the Linking AUdit and National datasets in Congenital HEart Services (LAUNCHES) project. Mortality, reintervention and hospital stay were described, and associated risk factors were explored using multivariable regression models for each of these three outcomes (Cox regression, Fine-Gray subdistribution hazard model and quantile regression at median, respectively).

**Results:**

A total of 3321 patients were included: n=669 (20.1%) had CoA with ventricular septal defect (VSD), n=331 (10.0%) had CoA with small VSD and n=2321 (69.9%) had isolated CoA. Mortality and cardiac reintervention at 10 years (from birth and CoA repair, respectively) were 3.7% (95% CI 3.0%; 4.4%) and 13.3% (12.1%; 14.5%), respectively. Compared with isolated surgical repair, isolated catheter repair (HR 3.7, (95% CI 2.2; 6)) and concomitant VSD closure (HR 1.34, (1; 1.9)) or pulmonary artery banding (HR 3.5, (2.4; 5.1)) had higher risk of reintervention. During the first year of life, the median time in hospital was 26 days (IQR 17; 44), decreasing to 1 (0; 2) day beyond 8 years. CoA with large VSD (−12, (−16; −8)), premature birth (−50, (−60; −40)), congenital comorbidity (−31, (−37; −25)), low weight (−23/kg, (−37; −11)) and younger age at first procedure (−6/year (−7; −5)) were associated with fewer days spent at home.

**Conclusions:**

Subgroups of patients with CoA are still at risk of unfavourable outcomes during childhood and adolescence follow-up, especially cardiac reintervention at a distance from initial repair. Hospital resource utilisation remains low beyond the first year of life in the majority of patients. Identified factors, while non-modifiable, remain useful in risk stratification and counselling.

**WHAT IS ALREADY KNOWN ON THIS TOPIC**

- While procedural outcomes and survival for corrected aortic coarctation are excellent, late cardiovascular comorbidities remain common.
- Modifiable factors, such as repair timing, associated defects and treatment choices, may influence the long-term course in adults with coarctation.

**WHAT THIS STUDY ADDS**

- Despite high survival rates into adolescence, reinterventions are frequent after coarctation repair in this group, and more common after catheter repair and in those with large ventricular septal defects.
- Hospital resource use significantly declines to a median of 1 inpatient day after the first year of life.
- Age at first procedure and comorbidities are key risk factors for mortality, reintervention and hospital stay duration.

**HOW THIS STUDY MIGHT AFFECT RESEARCH, PRACTICE OR POLICY**

- Modifiable factors, such as timing and treatment choices in childhood, may influence later cardiovascular complications, but larger linked studies such as this, with longer term follow-up, are needed to better integrate findings into practice.
- Identified non-modifiable risk factors can serve towards family and patient counselling, especially around hospital stay and reintervention planning.

## Introduction

 Coarctation of the aorta (CoA) is among the more common congenital heart diseases (CHDs), at an incidence of 4 out of 1000 live births.^1 2^ It can present as isolated CoA (including with aortic arch hypoplasia), in association with ventricular septal defects (CoA with VSDs), with patent arterial duct (PDA) or as part of complex CHD.^1^

In children with isolated CoA, the preferred treatment options are usually surgical,^2^,^4^ as opposed to adults where catheter-based management is preferred.^1^ Corrected isolated CoA has very good long-term survival, but reintervention and cardiovascular complications remain an issue, despite early repair and modern techniques.^5^,^7^ Factors associated with higher mortality and reintervention include low weight at intervention, need for repair <1 year of age, genetic syndromes or associated comorbidities and operation era.^8 9^ CoA with VSD is associated with higher early mortality,^10^,^12^ but no effect was described in late follow-up in multicentre registry data.^9^ Even if these are non-modifiable risk factors, better understanding of their role in late outcomes can improve clinical decision-making, treatment planning and family counselling, especially around reintervention timing and expectations.^2^

With mortality decreasing, indicators related to complications and morbidities become more important for healthcare providers, policymakers, patients and families.^2 6 12 13^ Despite low per-patient costs of childhood hospital care, through high prevalence, CoA ranks second only to single ventricle disease in total population healthcare costs.^14 15^ Understanding how these resources are distributed as inpatient, outpatient or intensive care, as well as throughout the child’s pathway to adult services, can inform both families and policymakers.

This study used the data infrastructure of the Linking AUdit and National datasets in Congenital HEart Services for Quality Improvement (LAUNCHES QI)^16^ project (England data) to address some of the current gaps in knowledge which exist in the management of isolated CoA (with and without VSD) in children by (1) describing the CoA treatment pathways and reparative procedure characteristics; (2) describing early and late hospital outcomes including mortality, reintervention and hospital resource utilisation; and (3) exploring risk factors associated with survival, reintervention and time spent at home in the first year of life.

## Methods

### National datasets and linkage

The National Congenital Heart Disease Audit (NCHDA) is the core dataset which was linked to the Paediatric Intensive Care Audit Network (PICANet) for patient admissions to paediatric intensive care units (ICUs); death registrations from the Office for National Statistics (ONS); and Hospital Episode Statistics (HES) routine administrative data on inpatient, outpatient and accident and emergency (A&E) care at hospitals in England. Linkage methodology and an overview of the dataset were described elsewhere.^16 17^ The LAUNCHES project received ethical approval from the Health Research Authority (reference: IRAS 246796) and the Confidentiality Advisory Group (reference: 18/CAG/0180).

### Patient selection and classification

All patients with a diagnosis of CoA (including aortic arch hypoplasia), with and without VSD (no other more complex associated defects), undergoing any cardiac procedure (surgical or interventional) between April 2000 and March 2017 were included. Bicuspid aortic valve was excluded if mention of stenosis (n=288). These are detailed in online supplemental figure 1.

Three CoA diagnosis subgroups were defined: isolated CoA, CoA with VSD (had either pulmonary PA banding or VSD closure on record) and CoA with small VSD (no VSD closure or PA banding on record, but diagnostic evidence of VSD).

All the procedures associated with a patient entry were defined as follows: pre-pathway if not part of the reparative procedure and occurred before correction; palliative procedure for PA banding done before CoA correction; treatment pathway procedures are those associated with reparative procedures, including staged CoA with VSD repair or with VSD closure done at a separate time; reinterventions (off pathway procedures) are any cardiac procedures performed after the CoA repair and not part of the treatment pathway, including recoarctation repair, VSD, PA, subaortic stenosis (SAS) relief, atrial septal defect (ASD) and other cardiac interventions.

Three reparative procedure types were defined: isolated CoA repair (surgery or transcatheter, and only CoA repair was done at the index procedure), CoA repair with VSD closure (VSD closure was done concomitantly to the CoA repair) and CoA repair with PA banding (PA band was placed at the time of CoA repair).

The corresponding diagnosis/procedure codes, specific procedure classifications,^18^ inclusion and exclusion criteria and diagnosis/procedure type classifications are further detailed in the online supplemental methods (and online supplemental tables 1–5).^17^

### Collected data and outcomes

All clinical data were organised in ‘care spells’, which can contain procedures, inpatient or intensive care stays, outpatient or emergency room (A&E) visits, in any combination, at no more than 1 day apart.^16 17^ The data extracted from the LAUNCHES QI dataset is detailed in online supplemental methods.

### Statistical analysis

Frequencies are given as numbers and percentages (%), all continuous values as median (IQR). Measures of effect are presented with 95% CIs unless otherwise stated. We applied data disclosure control rules throughout the manuscript, which suppress all counts from 1 to 5 (marked as *), allowing 0 to be shown. The number of records with non-missing values is provided for each reported value. Descriptive analyses of patient and procedure characteristics, hospital resource utilisation (days spent in hospital), reintervention and mortality were performed using the entire cohort, accounting for left truncation and right-censoring in each linked dataset. Mortality after birth was calculated using the Kaplan-Meier survival estimator up to the maximum follow-up age in ONS (right-censoring). The estimated probability of reintervention after coarctation repair was calculated using cumulative incidence functions up to the occurrence of competing events (death without reintervention) or maximum follow-up in NCHDA, whichever came earlier. Median hospital resource utilisation data are reported per successive year of follow-up (or month in the first year) using all patients with available data for at least part of each reported time period.

To evaluate risk factors for mortality, reintervention and days spent at home in the first year after CoA repair, a multivariable regression approach was used (Cox regression, Fine-Gray subdistribution hazard model and quantile regression at median, respectively), as detailed in online supplemental material.

All statistical analyses were conducted using the STATA/MP V.17.0 software (StataCorp LLC, College Station, Texas, USA) or R (R Core Team, 2014, Vienna, Austria). The Sankey diagram was created using SankeyMATIC (https://sankeymatic.com/).

## Results

A total of 3321 patients were included (online supplemental figure 1), of which 2321 (69.9%) had isolated CoA, 669 (20.1%) had CoA with VSD and 331 (10%) had CoA with small VSD. Table 1 provides detailed demographic, clinical and procedural data for the whole cohort.

**Table 1:** CoA patient group characteristics

Diagnosis	
Isolated coarctation	2321 (69.9%)
CoA with VSD	669 (20.1%)
CoA with small VSD	331 (10.0%)
Male	2025 (61.0%)
Ethnicity	
White	2704 (81.4%)
Black	99 (3.0%)
Asian	307 (9.2%)
Mixed/others	102 (3.1%)
Missing	109 (3.3%)
Area of residence deprivation	
Deprived area	
IMD Q1 (most deprived)	822 (24.8%)
IMD Q2	750 (22.6%)
Non-deprived area	
IMD Q3	627 (18.9%)
IMD Q4	563 (16.9%)
IMD Q5 (least deprived)	559 (16.8%)
Era (birth year in financial year)	
2000–2004	995 (30.0%)
2005–2008	748 (22.5%)
2009–2012	845 (25.4%)
2013–2016	733 (22.1%)
Prematurity (born <37 weeks gestation)	209 (6.3%)
Antenatal diagnosis	598 (18.0%)
Missing	512 (15.4%)
Congenital non-cardiac comorbidity*	218 (13.8%)
Low weight at first procedure (<2.5 kg)	312 (9.4%)
Acquired comorbidity at first procedure*	153 (9.7%)
Increased severity of illness at first procedure*	266 (16.9%)
Additional cardiac risk factor*	116 (7.4%)
Age at first procedure (days since birth)	
Median (IQR)	21 (9–104)
Neonate (age <30 days)	1848 (55.6%)
Infant (age ≤6 months and >=30 days)	831 (25.0%)
Infant (age >6 months) and older	640 (19.3%)

Definitions of risk factor terms described by Brown *et al*.^28^

Data are n (%) or median value (IQR).

Deprivation n=232 (7.0%) and low weight below 2.5 kg, n=118 (3.6%) included imputed data.

* Based on n=1578 patients who were born from 2009 onwards. Data quality for certain clinical variables was poor initially and improved after 2009 (when the processes for data quality were changed).

CoA, coarctation of the aorta; IMD, Indices of Multiple Deprivation; Q1–Q5, IMD quintiles; VSD, ventricular septal defect.

### CoA treatment pathways and reparative procedure characteristics

#### CoA treatment pathways

**Figure 1:**
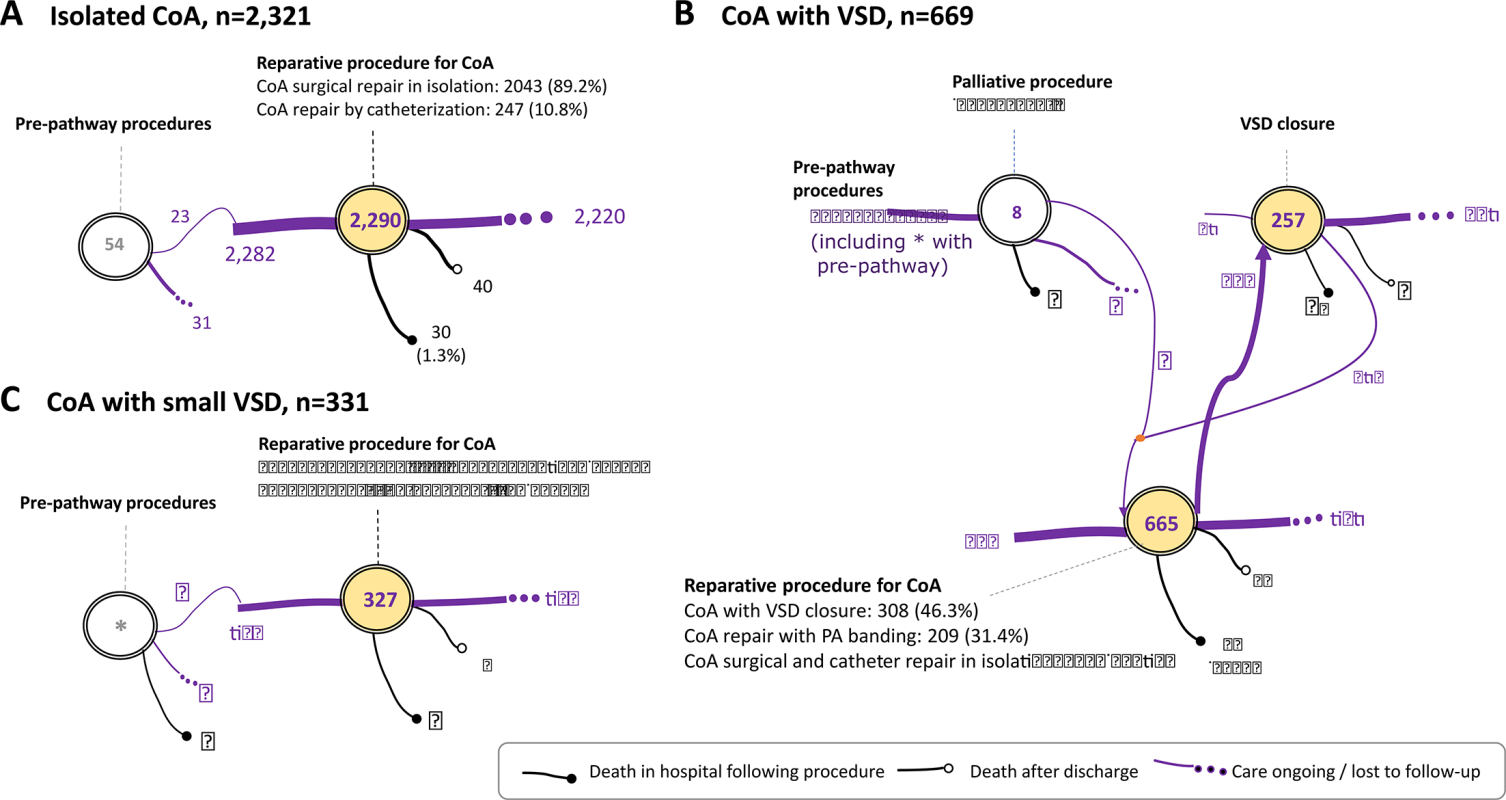
Planned treatment pathways and mortality events for patients with coarctation of the aorta (CoA). (A) Patients with isolated CoA. (B) Patients with CoA with ventricular septal defect (VSD). (C) Patients with CoA with small VSD. Most common pre-pathway procedure was patent arterial duct closure (34/60). Continuous black line with black circle end—death in hospital after a procedure; continuous black line with white circle end—death after discharge; continuous purple line—transition to care pathway procedure; interrupted purple line—ongoing care or lost to follow-up.

Treatment pathways for isolated CoA (figure 1), CoA with large VSD (figure 1B) and small VSD (figure 1C) highlight the impact of anatomical variants of management choices (further detailed in online supplemental table 6). Of the 148 patients with CoA and VSD who had an initial isolated CoA repair, 134 had a separate VSD closure and 14 only had a separate PA banding.

#### CoA reparative procedure characteristics

Proportion of surgical versus catheter repair, use of bypass and sternotomy, by diagnosis and CoA repair type are detailed in online supplemental table 7. The majority of surgical repair procedures for isolated CoA and CoA with small VSD were done without bypass (82.8% and 85.6%, respectively) and most used a thoracotomy (65.9% and 74.7%, respectively). In CoA patients with a VSD, catheter-based CoA repair was rare (6 cases). Bypass (58.3%) and sternotomy surgical (57.1%) approaches were favoured in this subgroup, reflecting the need for VSD closure or PA banding.

Age at CoA repair was lowest in CoA with VSD, followed by CoA with small VSD, and highest in isolated CoA. There was variability in CoA repair age between centres, with some showing higher upper age limits, but similar median values ranging from 12 to 33 days. The median age for CoA repair decreased between 2000 and 2016 from 45 to 14 days, with a more significant decrease in the upper age limit. Median age at CoA repair by diagnosis type, centre and year is shown in online supplemental table 8.

### Early and late outcomes in children with CoA

#### Early outcomes and ICU resource utilisation

Early (30-day), in-hospital and ICU outcomes, including for catheter CoA repair, by thoracotomy or bypass use are shown in online supplemental tables 9–14. Patients undergoing a catheter CoA repair had comparable 30-day and in-hospital mortality (0.4% and 1.5%, respectively), a higher likelihood or 30-day reintervention (4.3%) and shorter hospital length of stay (LoS) (median 2 day) with only a minority (n=26) having a recorded ICU stay. Patients having repair through thoracotomy and/or without bypass had lower early mortality and shorter ICU and hospital LoS compared with those needing sternotomy and/or bypass.

#### Late mortality through childhood and adolescence

The median follow-up was 12.2 (IQR 8.0, 17.1) years, and a maximum of 21.8 years. The overall 1-year and 5-year mortality rates for the cohort were 2.6% (2.0–3.1%) and 3.4% (2.7–4.0%), respectively (online supplemental figure 2A,B). This increases to 3.9% (3.2–4.6%) at the maximum follow-up of 21 years. The unadjusted 5-year mortality rate appeared to be higher but was not statistically significant for CoA with VSD, compared with CoA with small VSD and isolated CoA, at 5.5% (3.7–7.2%), 3.4% (1.4–5.4%) and 2.7% (2.1–3.4%), respectively. For the subgroup undergoing catheter-based CoA repair, death rates at 1, 5 and 10 years were 1.1% (0–2.4%), 2.3% (0.5%–4.1%) and 2.7% (0.7%–4.7%), respectively.

#### Cardiac reinterventions after CoA repair

**Figure 2:**
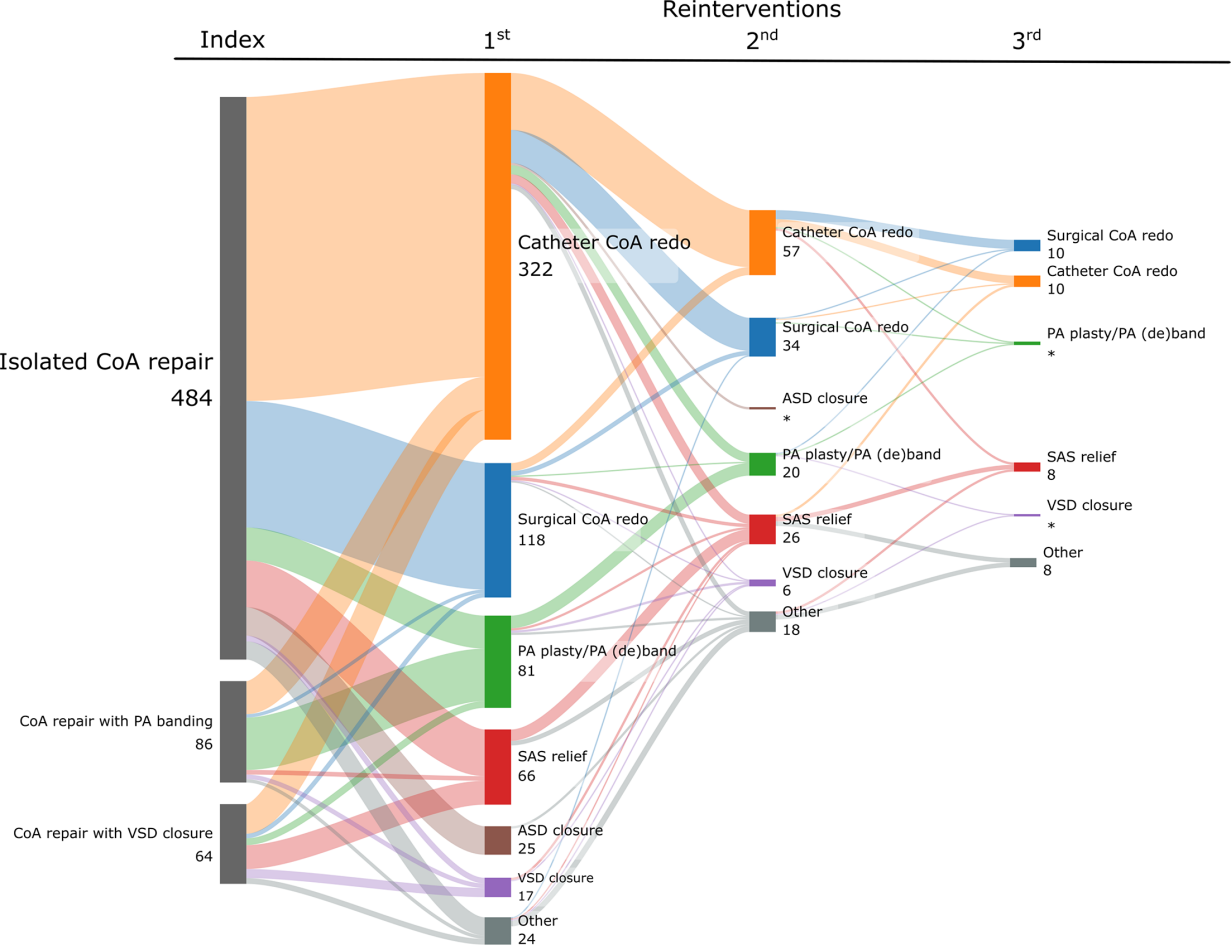
Sankey diagram showing reintervention types and sequence after coarctation of the aorta (CoA) repair, by index repair type. Patients with combined procedures are counted multiple times, within each reintervention category. Reinterventions later than third in sequence are not shown (n=14). Other reinterventions include electrophysiology, other valvular and unclassifiable miscellaneous procedures. Transparent bands and solid colour nodes reflect reinterventions of the same type within the sequence (orange—catheter recoarctation repair; blue—surgical recoarctation repair; green—PA arterioplasty or PA de(banding); red—SAS relief; brown—ASD closure; purple—VSD closure; grey—other). ASD, atrial septal defect; PA, pulmonary artery; SAS, subaortic stenosis; VSD, ventricular septal defect.

Reintervention data were available over a median follow-up of 5.4 (IQR 1.5–10.0) years and a maximum of 16.9 years. Among 3282 patients who underwent a coarctation repair, 634 (19.3%) experienced at least one reintervention, occurring at a median of 157 days (IQR 63–611) after the coarctation repair. Of patients who had a reintervention, 155 (4.7%) had multiple (≥2) reinterventions. The subtypes of reinterventions and their sequence are presented in figure 2, with the most common type being catheter recoarctation repair (stent or balloon), followed by surgical recoarctation repair and procedures related to the PA or PA de(banding).

**Figure 3:**
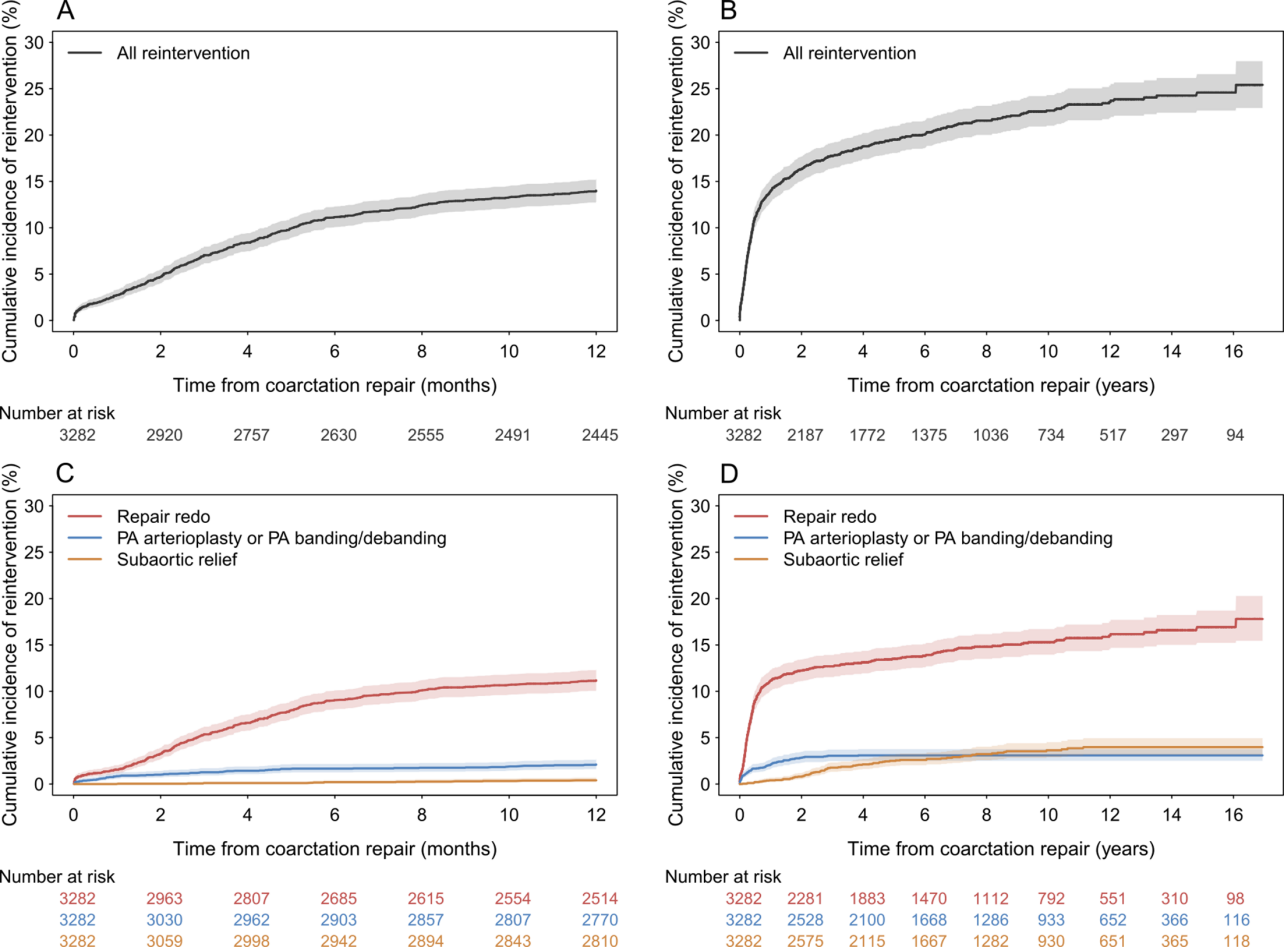
Cardiac reintervention after coarctation of the aorta (CoA) repair. (A) Cumulative incidence of reintervention in the first year of life for the whole cohort. (B) Cumulative incidence of reintervention at maximum follow-up for the whole cohort. (C) Cumulative incidence of reintervention in the first year of life by type. (D) Cumulative incidence of reintervention at maximum follow-up by type.

Any cardiac reintervention cumulative incidence increases steeply during the first year (figure 3A), when it reaches 14.0% (12.8–15.2%), then slowly afterwards until the maximum follow-up of 16 years (figure 3B). This steep increase is mostly due to recoarctation repair, with PA arterioplasty/PA de(banding) and SAS relief likelihood increasing later, in the first 2 years and after 2 years, respectively (figure 3C,D). The reintervention rates were higher within the subgroup undergoing catheter-based CoA repair, with cumulative incidences at 1, 5 and 10 years after CoA repair being 24.3% (19.1–29.9%), 42.0% (34.9–49.0 %) and 54.5% (45.6–62.6%), respectively.

#### Days spent in hospital from birth to 18 years old

**Figure 4:**
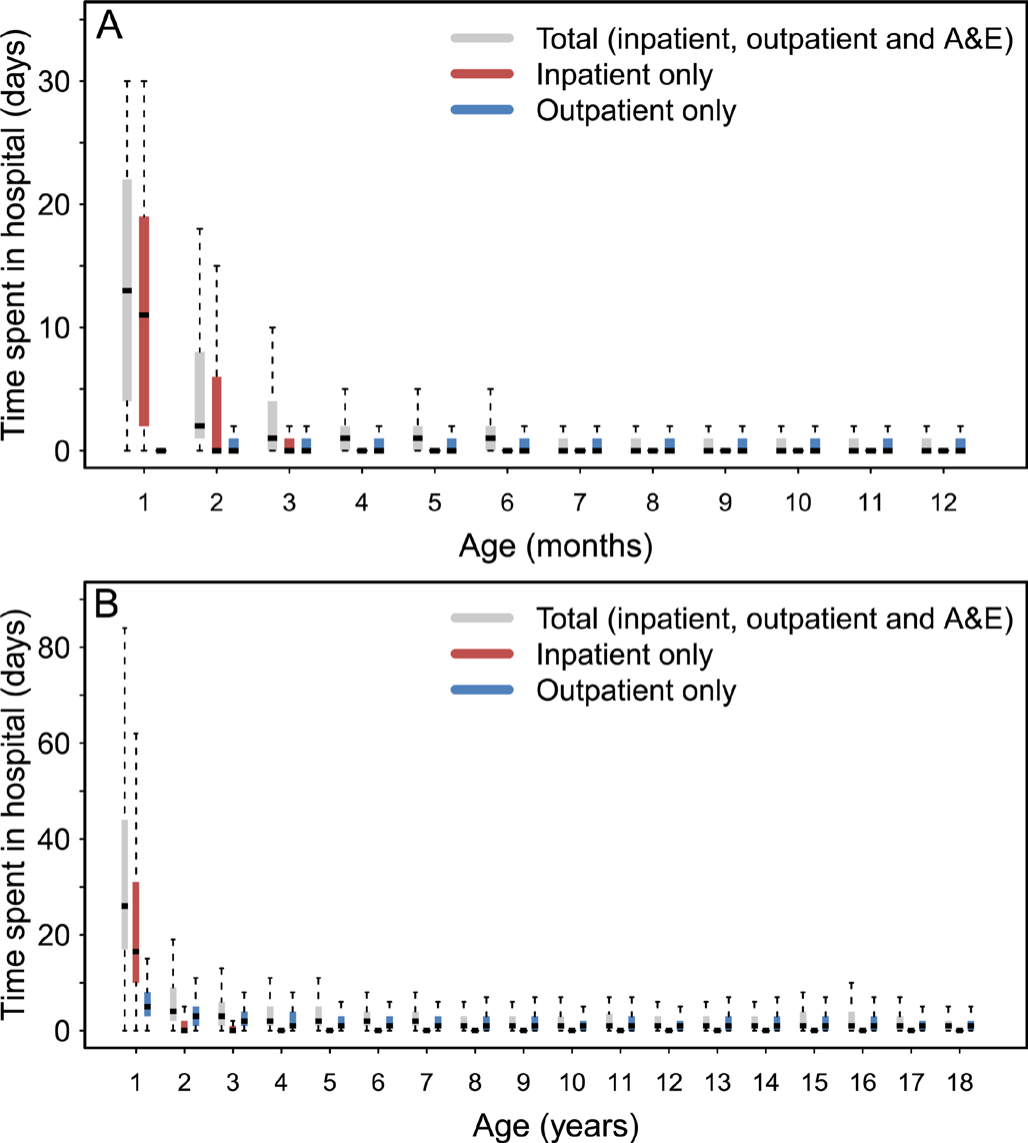
Days spent in hospital for patients with coarctation of the aorta (CoA) (A) during the first year of life and (B) in each year of follow-up from birth to 8 years old. After the age of 8 years, the median total days spent in hospital remains at 1 day/year. All panels show the median (horizontal black line), IQR (coloured solid bars) and 1.5 × IQR (dotted vertical lines). Outliers outside these limits are not shown.

The median number of days spent in hospital (inpatient, outpatient and A&E) per year was highest during the first year of life, at 26 (IQR 17–44) days, and decreased gradually down to 1 (IQR 0–2) day per year by the age of 18 years old (figure 4). Before 1 year of age, most of the days were spent as inpatient, at 16 (IQR 10–31) days. After that, inpatient days were uncommon. 71.9% of patients had >10 inpatient days in the first year, 6.1% in the second (when reinterventions were most common), 2.9% in the third, decreasing to <1% after the sixth year. After the sixth year, >80% of patients had no further inpatient days. Those undergoing catheter CoA repair spent at 4 days (IQR 1–13) in hospital during their first year of life overall, of which 3 (IQR 1–9) days inpatient and 1 (IQR 0–4) day outpatient.

### Risk factors associated with outcomes in patients with CoA

When adjusting for patient and procedural risk factors, type of CoA diagnosis was not significantly associated with survival, but associated comorbidities and younger age were (table 2). Neonatal age (binary variable) was not independently associated with late mortality independent of age as a continuous variable (p>0.5).

**Table 2:** Risk factors associated with late mortality in patients with CoA

Risk factors	Unadjusted HR (95% CI)	Adjusted HR (95% CI)
Diagnostic subtype		
Isolated CoA	Reference	Reference
CoA with VSD	1.80 (0.91 to 3.52)	0.93 (0.34 to 2.55)
CoA with small VSD	1.30 (0.66 to 2.57)	1.36 (0.50 to 3.70)
Additional cardiac risk factor	3.01 (1.81 to 5.01)***	3.57 (2.36 to 5.40)***
Prematurity	2.72 (1.69 to 4.36)***	2.52 (1.05 to 6.08)*
Congenital non-cardiac comorbidity	9.49 (6.11 to 14.73)***	8.19 (5.03 to 13.77)***
Low weight at first procedure	1.95 (1.03 to 3.70)*	0.81 (0.33 to 2.00)
Age at first procedure (years)*	0.50 (0.37 to 0.68)***	0.41 (0.21 to 0.84)*
Deprived area	1.87 (1.14 to 3.06)*	1.55 (0.73 to 3.29)

n=1559 patients included (born from 2009 onwards). n=19 had missing ethnicity data and were removed as possibly not missing at random (all survived). Death occurred in 44 patients (2.8%). Follow-up time: median 12.2 years (IQR 8.0–17.1, up to 21.8 years). Deprivation includes imputed data (6.4%).

Significance level (p value): 0.05*, 0.01**, 0.001***.

* Age at first procedures is equal to age at CoA repair in 97.7% of cases.

CoA, coarctation of the aorta; VSD, ventricular septal defect.

Patient level characteristics, choice of catheter repair, associated VSD or use of PA banding were risk factors associated with reinterventions (table 3). CoA with VSD diagnosis (but not small VSD) was associated with fewer days at home in the first year of life compared with isolated CoA, with other risk factors being detailed in table 4.

**Table 3:** Risk factors associated with late cardiac reintervention after CoA repair in multivariable analysis, by type of reintervention

Risk factors	Reintervention type			
	Any (n=261)	Recoarctation repair (n=172)	PA arterioplasty or PA (de)banding (n=37)	Subaortic stenosis relief (n=37)
CoA repair pathway				
Isolated CoA repair (surgical)	Reference	Reference	Reference	Reference
CoA repair by catheterisation	3.65 (2.23 to 5.96)***	4.76 (2.95 to 7.70)***	2.87 (0.65 to 12.66)	†
CoA repair with VSD closure	1.34 (0.95 to 1.88)	0.71 (0.36 to 1.42)	1.05 (0.38 to 2.91)	4.06 (1.78 to 9.27)***
CoA repair with PA banding	3.52 (2.44 to 5.09)***	1.41 (0.96 to 2.09)	18.97 (9.60 to 37.51)***	1.31 (0.33 to 5.15)
Additional cardiac risk factor	1.63 (1.04 to 2.54)*			
Preterm birth	1.61 (1.25 to 2.08)***	1.74 (1.17 to 2.61)**		
Congenital non-cardiac comorbidity			1.27 (0.85 to 1.88)	
Low weight at first procedure	1.28 (0.90 to 1.82)	1.38 (0.87 to 2.21)	1.65 (0.89 to 3.05)	
SoI marker at first procedure	1.59 (1.11 to 2.28)*	1.59 (1.09 to 2.32)*	1.85 (1.28 to 2.67)**	
Age at first cardiac procedure (days)			0.99 (0.98 to 1.00)	0.98 (0.97 to 0.99)***

Measure of effect is adjusted subdistribution HRs (sHRs) (with 95% CIs) for cardiac reintervention after CoA repair, by type of reintervention.

n=1563 patients included (born from 2009 onwards undergoing CoA repair).

Significance level (p value): 0.05*, 0.01**, 0.001***.

†N/A due to no occurrence.

CoA, coarctation of the aorta; PA, pulmonary artery; SoI, severity of illness; VSD, ventricular septal defect.

**Table 4:** Risk factors associated with median days spent at home in the first year of life after CoA repair

Risk factors	Days at home	
	Unadjusted	Adjusted
Diagnostic subtype		
Isolated CoA	Reference	Reference
CoA with VSD	−12 (−16, –8)***	−12 (−16, –8)***
CoA with small VSD	−2 (−4, 0)	−2 (−4, 0)
Male sex	3 (1, 5)**	−1 (−2, 1)
Deprived area	−2 (−4, 0)*	−2 (−4, 0)*
Birth year	−3 (−5, 0)*	−3 (−5, 0)*
2009–2012	Reference	Reference
2013–2016	−3 (−5, 0)*	−3 (−5, 0)*
Preterm birth	−50 (−60, –40)***	−31 (−41, –22)***
Additional cardiac risk factor	−6 (−11, –1)*	−6 (−11, –1)*
Congenital non-cardiac comorbidity	−31 (−37, –25)***	−26 (−31, –20)***
Low weight at first procedure	−23 (−37, –11)**	−23 (−36, –11)**
Acquired comorbidity at first procedure	−10 (−18, –2)*	−9 (−14, –3)***
SoI marker at first procedure	2 (−9, 0)*	−1 (−4, 2)
Age at first procedure (years)	6 (5, 7)***	5 (4, 6)***

Measure of effect is estimated difference in median days spent at home in the first year of life (with 95% CI).

n=1426 patients included (patients born from 2009 onwards, with inpatient data and not censored in before the age of 1 year). 23 patients who died before age 1 year old were assigned as 0 days at home. Median days at home was 339 days (IQR 318–347). Deprivation included imputed data (6.4%).

Significance level (p value): 0.05*, 0.01**, 0.001***.

CoA, coarctation of the aorta; SoI, severity of illness; VSD, ventricular septal defect.

Additional and detailed numerical data on mortality, reintervention and days-at-home analyses, complementing also figures3  4, are presented in online supplemental tables 15–18.

## Discussion

This study leverages the LAUNCHES^16^ project linked national database to evaluate early and late outcomes in paediatric CoA treatment, focusing on mortality, cardiac reinterventions and hospital stay along with associated risk factors. Despite excellent surgical results, findings reveal a significant reintervention burden during childhood and adolescence and late mortality that is higher than expected for a simple lesion in young people. Most patients had minimal hospital stays beyond the first year, although a subset with unmodifiable risk factors required more resources over time. Initial CoA management, such as timing, surgical versus catheter approach, and decisions on staged VSD repair, impacts outcomes across childhood and adolescence, and it will be important to determine if this carries over into influencing adult complications.

Multicentre or registry data on CoA treatment are few, but show similar early and late survival as those described in this more contemporary cohort.^9 11^ While late mortality is low when compared with other more complex defects, it is relatively high when considering these are children and adolescents, who would otherwise have low general mortality. Measures such as hospital stay and need for reinterventions are becoming increasingly important with improving survival. Isolated CoA was among the groups with the lowest total childhood healthcare costs per patient, but due to the high prevalence, it ranked second only to single ventricle in cumulative costs and hospitalisations.^14 15^ Using detailed national hospital episode statistics, we showed that time spent in hospital decreases greatly after the first 3 years of age. Most inpatient days are in the first year of life and beyond these are rare; after the sixth year, >80% of patients having no inpatient days. This favourable majority is nevertheless balanced by a smaller group of patients who experience more frequent hospital visits, at <1% after 6 years of age. It will be important to determine if this minority of children and adolescents with a higher burden of care become adults with late cardiovascular complications.^2 6 19 20^

The first step in this is identifying characteristics for these patients at risk. We were able to show that the main predictors for worse mortality, reintervention and hospital resource utilisation were additional cardiac and non-cardiac comorbidities, markers of severity of illness and prematurity. None of these are intrinsically modifiable but identifying high-risk patients early in the management pathway can lead to better surveillance of late complications and in turn improved care.^2^ Importantly, using these data for counselling, families of at-risk patients can better plan for time spent in hospital, reducing the impact on day-to-day life and adjusting expectations.

A more complex factor associated with worse mortality was younger age at repair, which is intrinsically linked to choice of treatment, especially when it comes to catheter-based CoA repair. Previous reports were not consistent in the effect reported for age of repair, but are also heterogeneous in design and definitions.^9 12 21^ In our study, however, age at first intervention remained a predictor independent of other associated risk factors, adding to current consensus.^2^ It is likely that the optimal intervention age should be decided on an individual basis, to allow for growth and development before surgery^2^ and can include palliative procedures such as balloon dilation or PA banding if VSD is associated,^22 23^ while controlling for the potential negative effects on cardiac function due to increased afterload.^6 23^ There is enthusiasm for complete anatomical repair at very early ages in most CHD, but these very early neonatal complex operations are still not clearly superior to delayed or staged approaches.^3 9 24^ We also found catheter CoA repair to be associated with significantly higher likelihood of re-coarctation repair, even if with lower mortality and first year of life hospital stay, when compared with surgical repair. This is a pattern that is emerging in other CHD as well, where catheter-based treatments trade-off days in hospital for later reinterventions.^25 26^ Whether this is due to patient or lesion characteristics contributing to the treatment choice, or a direct effect of the catheter intervention, it is difficult to establish retrospectively.

Another important factor influencing care in these patients is the association of VSD with CoA, the existing literature data being equivocal on its impact on outcomes.^10^,^12^ Part of the difficulty is that small and large defects will be managed differently, yet not all studies use the same classifications. In the current study, neither a small nor large VSD associated with CoA was found to have worse outcomes compared with isolated CoA. This is reassuring and suggests that the current treatment pathways are appropriately indicated and implemented.^2^ In terms of reintervention profile, we describe a higher risk of SAS relief procedures when VSD closure is performed as part of the CoA repair. This could be due to anatomical changes to the septum angle due to the VSD, as similar findings were described in recurrent SAS when VSD is associated, with or without CoA.^27^ Not surprisingly, a higher risk for PA procedures is seen (arterioplasty, de-banding and repeat band placement) when an initial PA band is placed at the time of CoA repair, but whether these have longer term impact on the incidence of PA stenoses is not known. Even if infrequent, SAS and PA reinterventions should be investigated and reported more accurately in CoA cohorts as these could become significant with further follow-up. The need for sternotomy and/or bypass was also found to be associated with higher early mortality and longer hospital LoS, but this will be confounded by the anatomical complexity of each case. Comparing treatment pathways was beyond the scope of this analysis but is nevertheless an important topic for future work.

### Limitations

This retrospective analysis is based on available data in linked national registries, so granular information on anatomical variants, imaging and clinical choice factors was not available. Patients with a CoA diagnosis but no intervention were not captured, but these are bound to be few and not reflect the same characteristics as a simple treated CoA cohort. Satisfactory classification in CoA with small or large VSD was possible based on timing of pathway procedures, whereas accurately identifying patients with arch hypoplasia could not be done with only the available diagnoses codes. We did not undertake a detailed analysis comparing the impact of surgical choices such as bypass use, sternotomy and VSD closure timings, as the clinical details that would inform these choices are not collected in the dataset. The choice between catheter and surgical CoA treatment can introduce patient selection bias, which might confound the analysis. Not all linked datasets had the same start date, and some variables were introduced at a later point; therefore, analyses had to be limited to subsets with complete data, detailed when relevant. Patients not undergoing at least one cardiac procedure could not be identified, so deaths without any treatment were not captured.

## Conclusions

In a national CoA cohort, subgroups of patients with CoA are still at risk of unfavourable outcomes during childhood and adolescence follow-up, especially cardiac reintervention at a distance from initial repair. In addition to recoarctation red-do procedures, SAS and PA reinterventions were also common. Risk factors associated with hospital resource utilisation in the first year of life are non-modifiable, but yet useful in risk stratification and informing future measures for improving quality of care.

## Supplementary material

10.1136/heartjnl-2024-325346 online supplemental file 1

## Data Availability

Data may be obtained from a third party and are not publicly available. All data relevant to the study are included in the article or uploaded as supplementary information.
