# Supplementary material for: Contemporary outcomes of childhood aortic coarctation interventions: a national registry analysis of mortality, reinterventions and hospital resource use
Source: Heart. 2025 Jul 8;112(4):e325346. doi: 10.1136/heartjnl-2024-325346 (PMC12911588; doi:10.1136/heartjnl-2024-325346)
Supplement: online supplemental file 1 [file heartjnl-112-4-s001.docx]

**Dorobantu et al.** Contemporary outcomes of childhood aortic coarctation interventions: a national registry analysis of mortality, reinterventions, and hospital resource use

**Supplementary material table of contents**

1. **Patient selection and statistical methodology**
   1. **Patient selection methodology**
   2. **Supplementary Figure 1**. Flowchart of included and excluded patient groups
   3. **Collected data and outcomes**
   4. **Statistical analysis – methodology of multivariable models**
2. **Diagnosis group and treatment pathway classification**
   1. **Supplementary Table 1**. Diagnosis and procedure included codes
   2. **Supplementary Table 2**. Exclusion Diagnostic codes
   3. **Supplementary Table 3**. NCHDA specific codes used for exclusions
   4. **Supplementary Table 4**. Diagnosis and procedure codes used to identify presence of VSD
   5. **Supplementary Table 5.** Procedure codes for SAS and ASD reinterventions
3. **Patient group and procedure characteristics**
   1. **Supplementary Table 6.** CoA repair types by diagnosis group
   2. **Supplementary Table 7**. CoA repair type (surgical, catheter), use of bypass and sternotomy by diagnosis type and procedure type
   3. **Supplementary Table 8**: Age at CoA repair by diagnosis type, hospital site, and year of birth
4. **Early outcomes and periprocedural ICU resource utilization**
   1. **Supplementary Table 9.** During CoA repair hospital spell
   2. **Supplementary Table 10.** For catheter-based CoA repair
   3. **Supplementary Table 11.** For surgical CoA repair with bypass
   4. **Supplementary Table 12.** For surgical CoA repair without bypass
   5. **Supplementary Table 13.** For surgical CoA repair with sternotomy
   6. **Supplementary Table 14.** For surgical CoA repair with thoracotomy
5. **Late outcomes, hospital utilization and associated risk factors**
   1. **Supplementary Table 15.** Cumulative incidence of death
   2. **Supplementary Table 16.** Cumulative incidence of cardiac reintervention
   3. **Supplementary Table 17.** Days spent in hospital during the first year of file and up 18 years old
   4. **Supplementary Table 18.** Risk factors associated with cardiac reintervention after CoA repair, by type of reintervention from unadjusted analysis

## **Patient Selection methodology**

We selected patients born between 1 April 2000 and 31 March 2017, having a simple coarctation of the aorta (CoA) diagnosis, with at least one cardiac procedure performed in in England or Wales to guarantee a complete intervention history using the LAUNCHES (Linking Audit and National datasets in Congenital Heart Services for Quality Improvement) dataset. A diagnosis of associated ventricular septal defect (VSD) was permitted. Patients born before April 2000 were excluded to ensure complete procedural history. Patients from overseas, Scotland and Northern Ireland were excluded as life status data from the Office of National Statistics does not cover these regions, only England and Wales. There were 39 patients (1.1%) who had no planned pathway procedures performed, 37 of these being censored and 2 dying with no CoA repair.

The automatic identification of CoA patients using the LAUNCHES dataset followed a three-step process, detailed in the following section. Briefly, first, we included all patients having a CoA diagnosis or a CoA repair procedure code, as detailed **Supplementary Table 1** or a NCHDA specific procedure classification for CoA repair (code number 47)(1) Then, we excluded complex CoA patients having any record with a exclusion code from **Supplementary Table 2** (diagnosis) and **Supplementary Table 3** (procedure), which uses the Partial Risk Adjustment in Surgery (PRAiS2) diagnosis categories,(2) and the NCHDA Specific Procedure categories for procedures. This resulted in 1695 patients being excluded due to more complex intracardiac defects being present (**Supplementary Figure 1**). Also excluded were 12 patients with a CoA diagnosis but no cardiac procedure on record and 33 patients with a CoA diagnosis, a VSD closure noted but no CoA repair on record (anomalous entries). The presence of a VSD was documented using the diagnosis or procedure codes in **Supplementary Table 4**.

**Supplementary Figure 1.** Flowchart of included and excluded patient groups

**
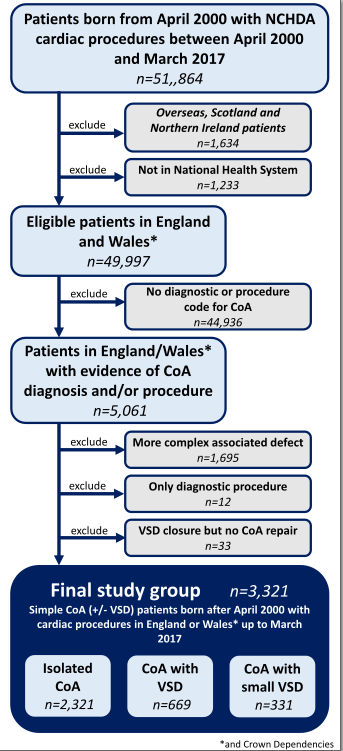
**

**Collected data and outcomes**

*Patient related data:* sex, ethnicity, area of residence deprivation index (IMD), preterm birth, antenatal diagnosis, and congenital comorbidity.

*Procedure related data:* age at procedure (first, CoA repair), weight at procedure, acquired comorbidity (the presence of an acquired complication related to CHD e.g. necrotising enterocolitis, renal failure), additional cardiac risk factors (associated cardiac conditions or complications not consisting of other CHD, such as pulmonary hypertension, cardiac dysfunction, cardiomyopathy), severity of illness marker (such as circulatory support, shock or acidosis),^16^ cardiac procedure type, procedure era and centre. These were previously defined as part of the Pediatric Cardiac Surgery (PRAiS) model.^16^

*Hospital resources utilization:* hospital length of stay (LoS) and ICU LoS for the CoA repair procedure spell, and from birth to end of follow-up: any and cardiac-related inpatient and ICU stays and outpatient or A&E department visits; peri-procedural ICU utilisation (ECMO, renal support, inotrope support, invasive/non-invasive ventilation),

*Mortality and reintervention:* in relation to the index CoA repair procedure (unless otherwise stated); early mortality and early cardiac reintervention (30 day and in-hospital), mid (1 and 5 years) and late (maximum follow-up) mortality and reintervention (any cardiac, surgical, transcatheter, and by anatomic subtype).

**Statistical analysis – methodology of multivariable models**

Frequencies are given as numbers and percentages (%), all continuous values as median (inter-quartile range). The number of records with non-missing values is provided for each reported value. Descriptive analyses of patient and procedure characteristics, hospital resource utilization (days spent in hospital), reintervention and mortality were performed using the entire cohort accounting for left truncation and right-censoring in each linked dataset. Mortality after birth was calculated using Kaplan-Meier survival estimator up to the maximum follow-up age in ONS (right-censoring). The estimated probability of reintervention after coarctation repair was calculated using cumulative incidence functions up to the occurrence of competing events (death without reintervention) or maximum follow-up in NCHDA, whichever came earlier. Median hospital resource utilization data is reported per successive year of follow-up (or month in the first year) using all patients with available data for at least part of each reported time period.

’Days at home’ before year one excluded any days spent in hospital including inpatient, outpatient and A&E visits. Patients who died before the first year were assigned as zero days at home as the worst outcome. Patients censored during the first year were removed from the analysis.^17^ The diagnosis subtype was the main risk factor of interest in the multivariable mortality and days at home models, whereas the main risk factor of interest in the reintervention model was the subtype of coarctation repair. For other candidate risk factors, those that were statistically significant (p<0.05) in univariable analysis were included in the multivariable models. Data quality for certain clinical variables (severity of illness [SoI], acquired comorbidities and congenital comorbidities) was only satisfactory after 2009 (when the processes for data quality changed).^15^ All models were therefore restricted to those patients born after 2009, so that we could include all relevant pre-procedural risk factors. We computed robust standard errors accounting for the clustering within each centre for all models.

## **Diagnosis group and treatment pathway classification**

**Step 1: Include patients who had evidence of CoA diagnosis or procedure**

Diagnostic code with evidence of primary diagnosis CoA/arch hypoplasia required (**Supplementary Table 1**) or specific procedure code for CoA repair (NCHDA specific procedure code number 47:coarctation_hypoplasia / Isolated coarctation/hypoplasia of aorta repair

**Step 2: Exclude patients who had more complex CHD**

Patients with any diagnostic code identified from **Supplementary Table 2** or procedure code identified from **Supplementary Table 3** were considered to have more complex associated congenital heart disease and were excluded. Patients undergoing heart transplant before CoA repair were also excluded.

**Step 3: Define CoA diagnosis types**

Three CoA diagnosis subgroups were defined: isolated CoA, CoA with VSD (had either pulmonary artery [PA] banding or VSD closure on record) and CoA with small VSD (no VSD closure or PA banding on record, but diagnostic evidence of VSD). Evidence of VSD was based on the diagnosis codes in **Supplementary Table 4**. The distinction between CoA with VSD and small VSD is relevant as the presence of a small VSD that does not require closure is clinically significant and can impact outcomes. A minority of the latter group with CoA repair close to the end of follow-up date could be misclassified due to delayed VSD closure not captured here, but this should not impact the analysis significantly.

**Step 4. Identify procedure pathways**

Treatment pathways were defined:

1. pre-pathway procedures performed before the first intervention that is part of the CoA repair pathway;

2. Palliative procedures are PA banding occurring before a CoA repair;

3. Reparative procedure for CoA which can include CoA repair, VSD closure and PA banding, in combination, as concomitant procedures or as staged procedures.

4. Reinterventions are procedures occurring after the final reparative pathway procedure.

Reparative procedure types were defined as:

1. CoA repair with VSD closure, which requires concomitant CoA repair procedure codes (**Supplementary table 1**) and VSD repair codes (**Supplementary Table 4**) for the same intervention.

2. CoA repair with PA banding which requires concomitant CoA repair procedure codes (**Supplementary table 1**) and the PA banding procedure code (121402: Pulmonary trunk band [PA band]).

3. Isolated CoA repair which permits neither concomitant VSD or PA band procedure codes.

Reinterventions are defined as:

1. CoA repair redo, catheter or surgical as any occurrence of CoA repair procedure code (**Supplementary Table 1**) after a repair procedure was already documented;

2. PA arterioplasty or PA (de)banding as any procedure following CoA repair that is either a surgical/catheter PA arterioplasty, placement of a PA band or surgical removal od a PA band; VSD closure reintervention occurs only if another VSD repair procedure or PA banding were noted as repair pathway procedure (concomitant or separate from CoA repair).

3. Subaortic stenosis (SAS) relief and atrial septal defect (ASD) reinterventions were identified using procedures codes listed in **Supplementary Table 5**.

| **Supplementary Table 1.** Diagnosis and procedure included codes |
| --- |
| **Diagnosis codes for CoA** |
| 092901. Aortic coarctation |
| 092911. Aortic arch hypoplasia (tubular) |
| 092944. Descending thoracic or abdominal aortic coarctation. |
| **Procedure codes for CoA** |
| 121800: Coarctation-hypoplasia of aorta repair |
| 121801: Aortic coarctation-hypoplasia repair by resection & end to end anastomosis |
| 121802: Aortic coarctation-hypoplasia repair by patch aortoplasty |
| 121803: Aortic coarctation-hypoplasia repair by subclavian flap aortoplasty |
| 121804: Balloon dilation of native aortic coarctation-hypoplasia |
| 121808: Balloon dilation of aortic recoarctation, |
| 121810: Aortic coarctation-hypoplasia repair by resection & extended end to end anastomosis |
| 121815: Aortic coarctation-hypoplasia repair by resection & insertion of tube graft |
| 121827: Aortic coarctation transluminal obstruction relief |
| 121830: Aortic arch repair |
| 121817. Stent placement at site of aortic coarctation, |
| 121822. Stent placement at site of aortic recoarctation, |
| 121827. Aortic coarctation transluminal obstruction relief, |
| 121848. Stent placement at site of native aortic coarctation-hypoplasia, |
| CoA, coarctation of the aorta; NCHDA, National Congenital Heart Disease Audit |

| **Supplementary table 2.** Exclusion Diagnostic codes |
| --- |
| 010101. Tetralogy of Fallot |
| 010102. Transposition of great arteries (concordant AV & discordant VA connections) & IVS |
| 010103. Congenitally corrected transposition of great arteries (discordant AV & VA connections) |
| 010104. Double outlet right ventricle |
| 010106. Pulmonary atresia + ventricular septal defect (VSD) (including Fallot type) |
| 010107. Pulmonary atresia with intact ventricular septum |
| 010109. Hypoplastic left heart syndrome |
| 010110.Transposition of the great arteries with concordant atrioventricular connections and ventricular septal defect. |
| 010114. Double inlet AV connection (double inlet ventricle) |
| 010116. Partially anomalous pulmonary venous connections: Scimitar syndrome |
| 010117. Double outlet right ventricle: Fallot type (subaortic or doubly committed VSD & pulmonary stenosis) |
| 010118. Double outlet right ventricle: transposition type (subpulmonary VSD) |
| 010119. Double outlet right ventricle with non-committed ventricular septal defect |
| 010120. AV septal defect and Tetralogy of Fallot |
| 010122. Functionally univentricular heart |
| 010124. Double outlet right ventricle: with intact ventricular septum |
| 010125. Pulmonary atresia + ventricular septal defect (VSD) + systemic-to-pulmonary collateral artery(ies) (MAPCA(s)) |
| 010126.Tetralogy of Fallot with pulmonary atresia. |
| 010133. Shone's syndrome: left heart obstruction at multiple sites, |
| 010140. Double outlet right ventricle with subaortic or doubly committed ventricular septal defect without pulmonary stenosis, ventricular septal defect type |
| 010157.Tetralogy of Fallot with pulmonary atresia and systemic-to-pulmonary collateral artery(ies) (MAPCA(s)). |
| 010309 AV and or/VA connections abnormal |
| 010403. Double inlet RV |
| 010404. Double inlet LV |
| 010405.Double inlet to solitary ventricle of indeterminate morphology. |
| 010501. Discordant VA connections (TGA) |
| 010503. Double outlet left ventricle |
| 020101.Extrathoracic heart (ectopia cordis). |
| 020303.Crisscross heart (twisted atrioventricular connections). |
| 020305. Solitary ventricle of indeterminate morphology |
| 030104. Right isomerism ('asplenia') |
| 030105. Left isomerism ('polysplenia') |
| 040600. Totally anomalous pulmonary venous connection: supracardiac |
| 040802.Congenital atresia of pulmonary vein(s). |
| 040804.Congenital anomaly of pulmonary vein(s). |
| 040805. Totally anomalous pulmonary venous connection |
| 040806. Obstructed pulmonary venous connection(s) |
| 040807. Anomalous pulmonary venous connection. |
| 040810. Totally anomalous pulmonary venous connection: intracardiac |
| 040820. Totally anomalous pulmonary venous connection: infracardiac |
| 040830. Totally anomalous pulmonary venous connection: mixed |
| 040831.Congenital pulmonary venous stenosis and-or hypoplasia. |
| 050201. Cor triatriatum (divided left atrium) |
| 050202. Supravalvar mitral ring |
| 050601. Common atrium (virtual absence of atrial septum) |
| 050603.Common atrium with common atrioventricular junction. |
| 060101. Triscuspid atresia |
| 060104. Tricuspid annular hypoplasia. |
| 060105. Overriding tricuspid valve. |
| 060107. Congenital tricuspid valvar stenosis. |
| 060111. Congenital anomaly of tricuspid valve. |
| 060126. Tricuspid atresia with absent valvar annulus (connection-junction). |
| 060134. Ebstein's malformation of tricuspid valve |
| 060201. Mitral atresia |
| 060202. Mitral atresia with imperforate mitral valve. |
| 060203.Dysplasia of mitral valve. |
| 060204. Mitral annular hypoplasia. |
| 060205. Overriding mitral valve. |
| 060207. Mitral valvar stenosis: congenital |
| 060211.Congenital anomaly of mitral valve. |
| 060213. Mitral subvalvar stenosis |
| 060221.Congenital anomaly of mitral subvalvar apparatus. |
| 060222. Congenital mitral subvalvar stenosis. |
| 060226. Mitral atresia with absent valvar annulus (connection-junction). |
| 060256. Parachute malformation of mitral valve |
| 060272. Congenital mitral valvar prolapse. |
| 060292. Mitral stenosis |
| 060293. Mitral valve stenosis |
| 060311.Congenital anomaly of right-sided atrioventricular valve in double inlet ventricle. |
| 060411.Congenital anomaly of left-sided atrioventricular valve in double inlet ventricle. |
| 060501. AVSD AV valvar abnormality |
| 060506. AVSD AV valvar regurgitation |
| 060514.Atypical common atrioventricular valve. |
| 060525. Double orifice of left ventricular component of common atrioventricular valve (left atrioventricular valve). |
| 060560.Common atrioventricular valvar regurgitation. |
| 060571.Atypical right ventricular component of common atrioventricular valve (right atrioventricular valve). |
| 060572.Atypical left ventricular component of common atrioventricular valve (left atrioventricular valve). |
| 060598. Deficient mural-lateral leaflet of left ventricular component of common atrioventricular valve (left atrioventricular vale) |
| 060600. Atrioventricular septal defect |
| 060601. AVSD: isolated atrial component (primum ASD)(partial) |
| 060608. AVSD: isolated ventricular component |
| 060609. AVSD: atrial & ventricular components with common AV orifice (complete) |
| 060610. AVSD: atrial & (restrictive) ventricular components + separate AV valves ('intermediate') |
| 060705. Atrioventricular septal defect (AVSD) with ventricular imbalance with dominant right ventricle and hypoplastic left ventricle. |
| 060706. Atrioventricular septal defect (AVSD) with ventricular imbalance with dominant left ventricle and hypoplastic right ventricle. |
| 060726. AVSD with ventricular imbalance |
| 060727. Atrioventricular septal defect (AVSD) with balanced ventricles. |
| 060728.Common atrioventricular junction with spontaneous fibrous closure of atrioventricular septal defect (AVSD). |
| 060736. Common atrioventricular valve with unbalanced commitment of valve to ventricles. |
| 060737.Common atrioventricular valve with unbalanced commitment of valve to right ventricle. |
| 060738.Common atrioventricular valve with unbalanced commitment of valve to left ventricle. |
| 070301. Double chambered right ventricle |
| 070520.Congenital right ventricular outflow tract obstruction. |
| 070530. Subpulmonary stenosis |
| 070532.Congenital subpulmonary stenosis. |
| 070841. Ventricular imbalance: dominant left ventricle + hypoplastic right ventricle |
| 070842. Ventricular imbalance: dominant right ventricle + hypoplastic left ventricle |
| 090101. Common arterial trunk (truncus arteriosus) |
| 090111. Common arterial trunk (truncus arteriosus) with aortic dominance and one pulmonary artery absent from trunk. isolated pulmonary artery. |
| 090112. Common arterial trunk (truncus arteriosus) with pulmonary dominance and aortic arch obstruction. |
| 090114. Common arterial trunk (truncus arteriosus) with aortic dominance and both pulmonary arteries arising from trunk. |
| 090115. Common arterial trunk (truncus arteriosus) with aortic dominance (no aortic arch obstruction). |
| 090118. Common arterial trunk (truncus arteriosus) with pulmonary dominance and interrupted aortic arch. |
| 090119. Common arterial trunk (truncus arteriosus) with pulmonary dominance and aortic coarctation. |
| 090200. Truncal valvar abnormality |
| 090201.Dysplasia of truncal valve. |
| 090203. Truncal valvar regurgitation |
| 090218.Congenital truncal valvar stenosis. |
| 090219.Congenital truncal valvar regurgitation. |
| 090401. Aortopulmonary window |
| 090407.Congenital aortopulmonary window. |
| 090501. Pulmonary valvar stenosis |
| 090504. Pulmonary valvar stenosis: congenital |
| 090505.Pulmonary 'annular' hypoplasia. |
| 090511. Pulmonary atresia |
| 090512. Pulmonary atresia: imperforate valve |
| 090516.Congenital pulmonary atresia. |
| 090525. Tetralogy of Fallot with absent pulmonary valve syndrome |
| 090592. Pulmonary stenosis |
| 090705. Absent or atretic pulmonary trunk (main pulmonary artery). |
| 090711. Pulmonary trunk hypoplasia |
| 090713. Supravalvar pulmonary trunk stenosis |
| 090715.Congenital supravalvar pulmonary stenosis. |
| 090716.Congenital anomaly of pulmonary arterial tree. |
| 090719. Congenital pulmonary trunk (main pulmonary artery) anomaly. |
| 090720. Congenital pulmonary trunk hypoplasia. |
| 090726. Solitary arterial trunk (absent intrapericardial pulmonary arteries) |
| 090902.Right pulmonary artery from arterial duct. |
| 090903.Right pulmonary artery from ascending aorta. |
| 090904.Left pulmonary artery from arterial duct. |
| 090905.Left pulmonary artery from ascending aorta. |
| 090908. Pulmonary artery from ascending aorta (hemitruncus) |
| 090911. Pulmonary artery from arterial duct. |
| 091001. Pulmonary arterial stenosis |
| 091006. Peripheral pulmonary arterial stenoses: at-beyond hilar bifurcation |
| 091007. Central pulmonary arterial stenosis: proximal to hilar bifurcation |
| 091010. Discontinuous (non-confluent) pulmonary arteries |
| 091025. Right pulmonary arterial stenosis |
| 091026. Left pulmonary arterial stenosis |
| 091027.Congenital pulmonary arterial branch stenosis. |
| 091028.Congenital right pulmonary arterial stenosis. |
| 091029. Congenital left pulmonary arterial stenosis. |
| 091030.Congenitally discontinuous. non-confluent right and left pulmonary arteries. |
| 091037.Congenital central pulmonary arterial stenosis or hypoplasia proximal to hilar bifurcation. |
| 091038. Congenital peripheral pulmonary arterial stenoses or hypoplasia at or beyond hilar bifurcation. |
| 091075.Absent or atretic right pulmonary artery. |
| 091077.Absent or atretic left pulmonary artery. |
| 091501. Aortic valvar stenosis: congenital |
| 091503. Aortic atresia |
| 091506. Aortic valvar atresia. |
| 091513. Aortic valvar stenosis |
| 091592. Aortic stenosis |
| 091600. Supravalvar aortic stenosis |
| 091618. Congenital supravalvar aortic stenosis. |
| 091701. Aortoventricular tunnel |
| 091702. Aorto - left ventricular tunnel |
| 092931. Interrupted aortic arch |
| 092932. Interrupted aortic arch distal to subclavian artery. type A. |
| 092933. Interrupted aortic arch between subclavian & common carotid arteries. type B. |
| 092934. Interrupted aortic arch between carotid arteries. type C. |
| 093134.Vascular ring of left aortic arch and right arterial duct or ligament. |
| 093135.Vascular ring of right aortic arch and left arterial duct or ligament. |
| 094101. Anomalous origin of coronary artery from pulmonary artery |
| 094103. Anomalous origin of left coronary artery from pulmonary artery (ALCAPA). |
| 094221.Anomalous aortic origin of coronary artery with ventriculo-arterial concordance. |
| 150503. Pulmonary vein obstruction |

| **Supplementary Table 3.** Procedure short codes and NCHDA specific codes used for exclusions | | |
| --- | --- | --- |
| **Short codes** | | |
| 120903: Damus-Kaye-Stansel type procedure: pulmonary trunk to aorta end/side anastomosis | | |
| 121004: Application of bilateral pulmonary arterial bands & transcatheter placement of stent in arterial duct | | |
| 121605: Balloon dilation of aortic valve | | |
| 121602: Aortic valvotomy: open | | |
| 120712: Left ventricular outflow tract obstruction relief: complex (Konno etc) | | |
| 122300: Anomalous coronary artery (eg ALCAPA) repair | | |
| 120806: Ventricular septal defect (VSD) enlargement | | |
| 121629: Aortic valvar replacement using mechanical prosthesis | | |
| 123103: Modified right Blalock interposition shunt | | |
| 123146:Modified Blalock interposition shunt | | |
| 123720: Double lung transplant | | |
| 122920: Double outlet right ventricle repair | | |
| 120157: Atrial baffle procedure | | |
| 121604: Aortic valvotomy: closed | | |
| 121663: Aortic root replacement using homograft | | |
| 120605: Balloon dilation of right ventricular outflow tract | | |
| 123134: Occlusion of systemic-to-pulmonary arterial shunt by transluminal device-embolus | | |
| 123119: Balloon dilation of systemic-to-pulmonary arterial shunt | | |
| 121302: Pulmonary valvotomy: open | | |
| 120835: Supravalvar aortic stenosis repair | | |
| 120002: Partially anomalous pulmonary venous connection repair | | |
| 120400: Atrioventricular septal defect procedure | | |
| **NCHDA Specific Procedure codes** | | |
| 01:norwood | 33:pulmonary_atresia_vsd | |
| 04:common_arterial_trunk_aorta_repair | 34:mapca_unifocalisation | |
| 05:common_arterial_trunk_repair | 35:absent_pulmonary_valve_syndrome | |
| 06:cctga_repair_a | 36:fallot | |
| 07:cctga_repair_b | 37:rv_pa_conduit | |
| 08:atrial_switch | 38:vsd_rvoto | |
| 09:rastelli_rev | 39:supra_valvar_aortic_stenosis | |
| 10:transposition_complex | 41:ap_window | |
| 11:transposition_arch | 42:anomalous_coronary | |
| 12:transposition_vsd | 43:cor_triatriatum | |
| 13:transposition | 45:arterial_shunt | |
| 14:tapvc_shunt | 46:iaa | |
| 15:tapvc | 48:pulmonary_vein_stenosis | |
| 16:fontan | 49:conduit_replacement | |
| 17:glenn | 58:pda_stent | |
| 18:avsd_fallot_a | 59:pulmonary_valve_replacement_transluminal | |
| 19:avsd_fallot_b | 60:rvot_stent | |
| 20:avsd_complete | 61:pulmonary_valve_radiofrequency | |
| 21:avsd_partial | 62:blade_atrial_septostomy | |
| 22:mitral_valve_replacement | 63:balloon_atrial_septostomy | |
| 23:ross_konno_a | 64:pulmonary_vein_intervention | |
| 24:ross_konno_b | 69:balloon_pulmonary_valve | |
| 25:ross | 74:pa_stent | |
| 26:aortic_root_replacement | 75:pa_ballooning | |
| 27:aortic_valve_replacement | 76:mapca_transluminal | |
| 28:tricuspid_valve_replacement | 77:conduit_balloon_stent | |
| 29:pulmonary_valve_replacement |  | |
| **Supplementary table 4.** Diagnosis and procedure codes used to identify presence of ventricular septal defect (VSD) | |  |
| Diagnosis codes | |  |
| 071000. VSD | |  |
| 071001. Perimembranous VSD | |  |
| 071012. VSD + malaligned outlet septum | |  |
| 071101. Muscular VSD | |  |
| 071200. Subarterial VSD | |  |
| 071201. Doubly committed subarterial VSD | |  |
| 071402. Communication between left ventricle + right atrium (Gerbode defect) | |  |
| 071405. Inlet VSD | |  |
| 071501. Tiny VSD (Maladie de Roger) | |  |
| 071504. Multiple VSDs | |  |
| 071505. Single VSD | |  |
| Procedure codes | |  |
| 120801. Ventricular septal defect (VSD) closure, | |  |
| 120802. Ventricular septal defect (VSD) closure by direct suture, | |  |
| 120803. Ventricular septal defect (VSD) closure using patch, | |  |
| 120807. Ventricular septal defect (VSD) closure with transluminal device, | |  |
| 120816. Closure of multiple ventricular septal defect (VSD)s, | |  |
| 120828. Intraoperative ventricular septal defect (VSD) closure with transluminal  device (hybrid approach), | |  |

| **Supplementary Table 5.** Procedure codes for SAS and ASD reinterventions |
| --- |
| Subaortic stenosis (SAS) relief |
| 120700.Left ventricular outflow tract procedure |
| 120701. Subaortic fibromuscular shelf resection |
| 120711. Left ventricular outflow tract myectomy-myotomy |
| 120713.Left ventricular outflow tract obstruction relief |
| 120822. Subaortic obstruction relief |
| Atrial septal defect (ASD) closure |
| 120101. ASD closure |
| 120102. ASD closure with direct suture |
| 120103. ASD closure with patch |
| 120106.Atrial septal defect (ASD) secundum closure with transluminal device |
| 120110. Sinus venosus ASD closure |

| **Supplementary Table 6.** CoA repair types by diagnosis group | | | | | |
| --- | --- | --- | --- | --- | --- |
|  | **CoA repair (all types)** | **Surgical isolated CoA repair** | **Catheter isolated CoA repair** | **CoA repair with concomitant VSD closure** | **CoA repair with PA banding** |
| **Whole cohort (n=3,321)** | | | | | |
| n (%) | 3,282  (98.8%) | 2,498  (75.2%) | 267  (8.0%) | 308  (9.3%) | 209  (6.3%) |
| Age (days) | 20  (9-101) | 19  (9-86) | 1,456  (363-3,057) | 14  (7-41) | 11  (7-17) |
| **Isolated CoA (n=2,321)** | | | | | |
| n (%) | 2,290  (98.7%) | 2,043  (88.0%) | 247  (10.7%) |  |  |
| Age (days) | 33  (9-190) | 33  (9-190) | 1,532  (483-3,132) |  |  |
| **CoA with VSD (n=669)** | | | | | |
| n (%) | 665  (99.4%) | 143  (21.4%) | * | 308  (46.0%) | 209  (31.2%) |
| Age (days) | 12  (7-30) | 11  (6-33) | 17  (17-133) | 14  (7-41) | 11  (7-17) |
| **CoA with small VSD (n=331)** | | | | | |
| n (%) | 327  (98.8%) | 312  (94.3%) | 15  (4.5%) |  |  |
| Age (days) | 16  (9-59) | 15  (9-53) | 229  (106-1491) |  |  |
| *CoA, coarctation of the aorta; VSD, ventricular septal defect.* | | | | | |

| **Supplementary Table 7**. CoA repair type (surgical, catheter), use of bypass and sternotomy by diagnosis type and procedure type. | | | | | | | |
| --- | --- | --- | --- | --- | --- | --- | --- |
|  | **Intervention type** | | **Bypass (if surgical)** | | **Sternotomy (if surgical)** | | |
|  | **Surgical** | **Catheter** | **Yes** | **No** | **Yes** | **No** | **Missing** |
| **Total** | 3,015  (91.9) | 267  (8.1) | 780  (25.9) | 2,235  (74.1) | 833  (27.6) | 1,738  (57.6) | 444  (14.8) |
| **Diagnosis type** | | | | | | | |
| Isolated CoA | 2043  (89.2) | 247  (10.8) | 351  (17.2) | 1,692  (82.8) | 393  (19.2) | 1,302  (65) | 348  (17.0) |
| CoA with small VSD | 312  (95.4) | 15  (4.6) | 45  (14.4) | 267  (85.6) | 64  (20.5) | 230  (73.7) | 18  (5.8) |
| CoA with VSD | 660  (99.1) | * | 384  (58.2) | 276  (41.8) | 376  (57.0) | 206  (31.2) | 76  (11.8) |
| **Procedure type** | | | | | | | |
| Isolated CoA repair | 2,498  (90.3) | 267  (9.7) | 407  (16.3) | 2,091  (83.7) | 474  (19) | 1,625  (65) | 399  (16) |
| CoA repair with PA band | 209  (100) | 0  (0) | 66  (31.6) | 143  (68.4) | 70  (33.5) | 112  (53.6) | 27  (12.9) |
| CoA repair with VSD closure | 308  (100) | 0  (0) | 307  (100) | * | 289  (94.1) | * | 18  (5.8) |
| *CoA, coarctation of the aorta; VSD, ventricular septal defect.* | | | | | | | |

| **Supplementary Table 8**: CoA repair by diagnosis type, hospital site, and year of birth. | | |
| --- | --- | --- |
|  | **Patients (n)** | **Age at repair median (IQR)** |
| By diagnosis type | | |
| Isolated CoA | 2290 | 33 (9-190) |
| CoA with VSD | 665 | 12 (7-30) |
| CoA with small VSD | 327 | 16 (9-59) |
| By hospital site | | |
| A | 509 | 28 (12-112) |
| B | 390 | 12 (6-57) |
| C | 365 | 24 (9-193) |
| D | 355 | 12 (4-89) |
| E | 353 | 33 (12-140) |
| F | 342 | 20 (9-70) |
| G | 300 | 22 (9-103) |
| H | 229 | 24 (10-83) |
| I | 222 | 19 (9-75) |
| J | 190 | 28 (11-114) |
| K | 64 | 35 (9-98) |
| By birth year (financial year) | | |
| 2000 | 214 | 33 (11-367) |
| 2001 | 198 | 45 (11-288) |
| 2002 | 197 | 29 (9-412) |
| 2003 | 188 | 25 (11-331) |
| 2004 | 198 | 41 (9-212) |
| 2005 | 178 | 32 (11-234) |
| 2006 | 183 | 17 (9-112) |
| 2007 | 204 | 26 (9-98) |
| 2008 | 183 | 22 (8-142) |
| 2009 | 202 | 18 (9-94) |
| 2010 | 199 | 17 (8-89) |
| 2011 | 206 | 21 (9-88) |
| 2012 | 238 | 19 (8-67) |
| 2013 | 189 | 15 (7-42) |
| 2014 | 206 | 15 (8-70) |
| 2015 | 179 | 14 (8-60) |
| 2016 | 159 | 16 (8-36) |
| *CoA, coarctation of the aorta; IQR, inter-quartile range; VSD, ventricular septal defect.* | | |

| **Supplementary Table 9.** Early outcomes and periprocedural ICU resource utilization during CoA repair hospital spell | | |
| --- | --- | --- |
| **Outcome** | **% or duration in days** | **Reference group size** |
| 30 day mortality, n (%)^a^ | 32 (1.0%) | n=3,136 |
| In-hospital mortality, n (%) | 49 (1.5%) | n=3,282 |
| 30 day cardiac reintervention, n (%)^b^ | 87 (2.8%) | n=3,112 |
| ICU resource utilization^c^ |  |  |
| ECMO used | 10 (0.3%) | n=1,556 |
| ECMO duration | 8 (5-9) | n=10 |
| Renal support used | 61 (3.9%) | n=1,556 |
| Renal support duration | 4 (2-10) | n=61 |
| Inotrope support used | 1,262 (81.1%) | n=1,556 |
| Inotrope support duration | 4 (2-7) | n=1,262 |
| Invasive ventilation in ICU | 2,458 (98.0%) | n=2,508 |
| Invasive ventilation duration | 3 (2-6) | n=2,458 |
| Non-invasive ventilation in ICU | 1,650 (96.7%) | n=1,708 |
| Non-invasive ventilation duration | 3 (2-7) | n=1,650 |
| ICU length of stay^c^ |  |  |
| Total (days) | 3 (2-6) | n=2,580 |
| Pre-repair | 1 (0-4) | n=2,580 |
| Post-repair | 2 (1-4) | n=2,580 |
| Hospital length of stay^d^ |  |  |
| Total (days) | 11 (7-21) | n=3,069 |
| Pre-repair | 3 (1-6) | n=3,069 |
| Post-repair | 7 (5-13) | n=3,069 |
| *ECMO, extracorporeal mechanical oxygenation; ICU, intensive care unit; IQR, inter-quartile range; CoA, coarctation of the aorta.*  *Proportions are based on patients with available data and expressed in %. Duration of ICU support measures and length of stay are calculated based on subgroup where each type was used/reported and expressed as median (IQR).*  *^a^ exclusion: n=146 censored with less than 30 days after repair, including n=1 patient with missing discharge age*  *^b^ exclusion: n=25 died before 30 days without a reintervention; additionally, n=145 censored with no reintervention and less than 30 days follow-up after repair.*  *^c^ ICU data were available in n=2,580 (78.6%) of CoA repair spells.*  *^d^ exclusion: n=206 had no linkage to in-patient data; n=1 missing age at discharge; n=7 had linkage errors* | | |

| **Supplementary Table 10.** Early outcomes and periprocedural intensive care unit (ICU) resource utilization in patients undergoing catheter based coarctation (CoA) repair | | |
| --- | --- | --- |
| **Outcome** | **Size of reference patient group** | **% or duration in days)** |
| 30 day mortality, n (%)^a^ | n=256 | 1 (0.4) |
| In-hospital mortality, n (%) | n=267 | 3 (1.5) |
| 30 day cardiac reintervention, n (%)^b^ | n=253 | 11 (4.3) |
| ICU length of stay^c^ |  |  |
| Total (days) | n=22 | 8 (3-22) |
| Pre-repair | n=22 | 1 (1-1) |
| Post-repair | n=22 | 7 (1-16) |
| Hospital length of stay^d^ |  |  |
| Total (days) | n=251 | 2 (1-2) |
| Pre-repair | n=251 | 1 (1-1) |
| Post-repair | n=251 | 1 (1-1) |
| *Proportions are based on patients with available data and expressed in %. Duration of ICU support measures and length of stay are calculated based on subgroup where each type was used/reported and expressed as median (IQR).*  *^a^ exclusion: n=10 censored in NCHDA with less than 30 days after repair (no linkage to ONS so censored at their maximum discharge age)*  *^b^ exclusion: n=1 died before 30 days without a reintervention; additionally, n=13 censored in NCHDA with no reintervention and less than 30 days follow-up after repair.*  *^c^ PICANet data were available in n=26 (9.4%) of CoA repair by catheterization spells.*  *^d^ exclusion: n=16 had no linkage to in-patient data.* | | |

| **Supplementary Table 11.** Early outcomes and periprocedural intensive care unit (ICU) resource utilization in patients undergoing catheter based coarctation (CoA) repair using surgical approach with cardiopulmonary bypass (n=780) | | |
| --- | --- | --- |
| **Outcome** | **Size of reference patient group** | **% or duration in days)** |
| 30 day mortality, n (%)^a^ | n=745 | 16 (2.1) |
| In-hospital mortality, n (%) | n=780 | 23 (3.0) |
| 30 day cardiac reintervention, n (%)^b^ | n=732 | 23 (3.1) |
| ICU length of stay^c^ |  |  |
| Total (days) | n=725 | 5 (3-8) |
| Pre-repair | n=725 | 1 (0-2) |
| Post-repair | n=725 | 4 (2-6) |
| Hospital length of stay^d^ |  |  |
| Total (days) | n=731 | 17 (11-29) |
| Pre-repair | n=731 | 4 (2-8) |
| Post-repair | n=731 | 11 (7-20) |
| *Proportions are based on patients with available data and expressed in %. Duration of ICU support measures and length of stay are calculated based on subgroup where each type was used/reported and expressed as median (IQR).*  *^a^ exclusion: n=35 censored in NCHDA with less than 30 days after repair (no linkage to ONS so censored at their maximum discharge age)*  *^b^ exclusion: n=13 died before 30 days without a reintervention; additionally, n=35 censored in NCHDA with no reintervention and less than 30 days follow-up after repair.*  *^c^ PICANet data were available in n=725 (92.9%) of CoA repair using bypass spells.*  *^d^ exclusion: n=49 had no linkage to in-patient data or had missing discharge age in the spell.* | | |

| **Supplementary Table 12.** Early outcomes and periprocedural intensive care unit (ICU) resource utilization in patients undergoing catheter based coarctation (CoA) repair using surgical approach without cardiopulmonary bypass (n=2,235) | | |
| --- | --- | --- |
| **Outcome** | **Size of reference patient group** | **% or duration in days)** |
| 30 day mortality, n (%)^a^ | n=2,134 | 15 (0.7) |
| In-hospital mortality, n (%) | n=2,235 | 23 (1.0) |
| 30 day cardiac reintervention, n (%)^b^ | n=2,123 | 53 (2.5) |
| ICU length of stay^c^ |  |  |
| Total (days) | n=1,829 | 2 (2-5) |
| Pre-repair | n=1,829 | 1 (0-1) |
| Post-repair | n=1,829 | 1 (1-3) |
| Hospital length of stay^d^ |  |  |
| Total (days) | n=2,090 | 11 (7-18) |
| Pre-repair | n=2,090 | 3 (1-6) |
| Post-repair | n=2,090 | 7 (5-11) |
| *Proportions are based on patients with available data and expressed in %. Duration of ICU support measures and length of stay are calculated based on subgroup where each type was used/reported and expressed as median (IQR).*  *^a^ exclusion: n=101 censored in NCHDA with less than 30 days after repair (no linkage to ONS so censored at their maximum discharge age)*  *^b^ exclusion: n=11 died before 30 days without a reintervention; additionally, n=101 censored in NCHDA with no reintervention and less than 30 days follow-up after repair.*  *^c^ PICANet data were available in n=1,829 (81.9%) of CoA repair (using non-bypass) spells.*  *^d^ exclusion: n=155 had no linkage to in-patient data or had missing discharge age in the spell.* | | |

| **Supplementary Table 13.** Early outcomes and periprocedural intensive care unit (ICU) resource utilization in patients undergoing catheter based coarctation (CoA) repair using surgical approach with sternotomy (n=833) | | |
| --- | --- | --- |
| **Outcome** | **Size of reference patient group** | **% or duration in days)** |
| 30 day mortality, n (%)^a^ | n=795 | 14 (1.8) |
| In-hospital mortality, n (%) | n=833 | 20 (2.4) |
| 30 day cardiac reintervention, n (%)^b^ | n=784 | 22 (2.8) |
| ICU length of stay^c^ |  |  |
| Total (days) | n=807 | 5 (2-8) |
| Pre-repair | n=807 | 1 (0-2) |
| Post-repair | n=807 | 3 (1-6) |
| Hospital length of stay^d^ |  |  |
| Total (days) | n=785 | 16 (10-28) |
| Pre-repair | n=785 | 4 (2-8) |
| Post-repair | n=785 | 11 (7-18) |
| *Proportions are based on patients with available data and expressed in %. Duration of ICU support measures and length of stay are calculated based on subgroup where each type was used/reported and expressed as median (IQR).*  *^a^ exclusion: n=38 censored in NCHDA with less than 30 days after repair (no linkage to ONS so censored at their maximum discharge age)*  *^b^ exclusion: n=11 died before 30 days without a reintervention; additionally, n=38 censored in NCHDA with no reintervention and less than 30 days follow-up after repair.*  *^c^ PICANet data were available in n=807 (96.9%) of CoA repair (using sternotomy) spells.*  *^d^ exclusion: n=48 had no linkage to in-patient data or had missing discharge age in the spell.* | | |

| **Supplementary Table 14.** Early outcomes and periprocedural intensive care unit (ICU) resource utilization in patients undergoing catheter based coarctation (CoA) repair using surgical approach with thoracotomy (n=1,738) | | |
| --- | --- | --- |
| **Outcome** | **Size of reference patient group** | **% or duration in days)** |
| 30 day mortality, n (%)^a^ | n=1,684 | 10 (0.6) |
| In-hospital mortality, n (%) | n=1,738 | 16 (0.9) |
| 30 day cardiac reintervention, n (%)^b^ | n=1,676 | 39 (2.3) |
| ICU length of stay^c^ |  |  |
| Total (days) | n=1,665 | 3 (1-5) |
| Pre-repair | n=1,665 | 1 (0-1) |
| Post-repair | n=1,665 | 1 (1-3) |
| Hospital length of stay^d^ |  |  |
| Total (days) | n=1,656 | 11 (7-18) |
| Pre-repair | n=785 | 3 (1-6) |
| Post-repair | n=785 | 7 (5-11) |
| *Proportions are based on patients with available data and expressed in %. Duration of ICU support measures and length of stay are calculated based on subgroup where each type was used/reported and expressed as median (IQR).*  *^a^ exclusion: n=54 censored in NCHDA with less than 30 days after repair (no linkage to ONS so censored at their maximum discharge age)*  *^b^ exclusion: n=8 died before 30 days without a reintervention; additionally, n=54 censored in NCHDA with no reintervention and less than 30 days follow-up after repair.*  *^c^ PICANet data were available in n=1,665 (95.7%) of CoA repair (non-sternotomy) spells.*  *^d^ exclusion: n=84 had no linkage to in-patient data or had missing discharge age in the spell.* | | |

**Supplementary Figure 2.** Mortality during childhood, adolescence and early adulthood in patients with CoA. **A.** Cumulative incidence of death in the first year of life for the whole cohort. **B.** Cumulative incidence of death at maximum follow-up for the whole cohort. **C.** Cumulative incidence of death in the first year of life by CoA diagnosis subtypes. **D.** Cumulative incidence of death at maximum follow-up by CoA diagnosis type. Numerical values detailed in **Supplementary Table 15.**


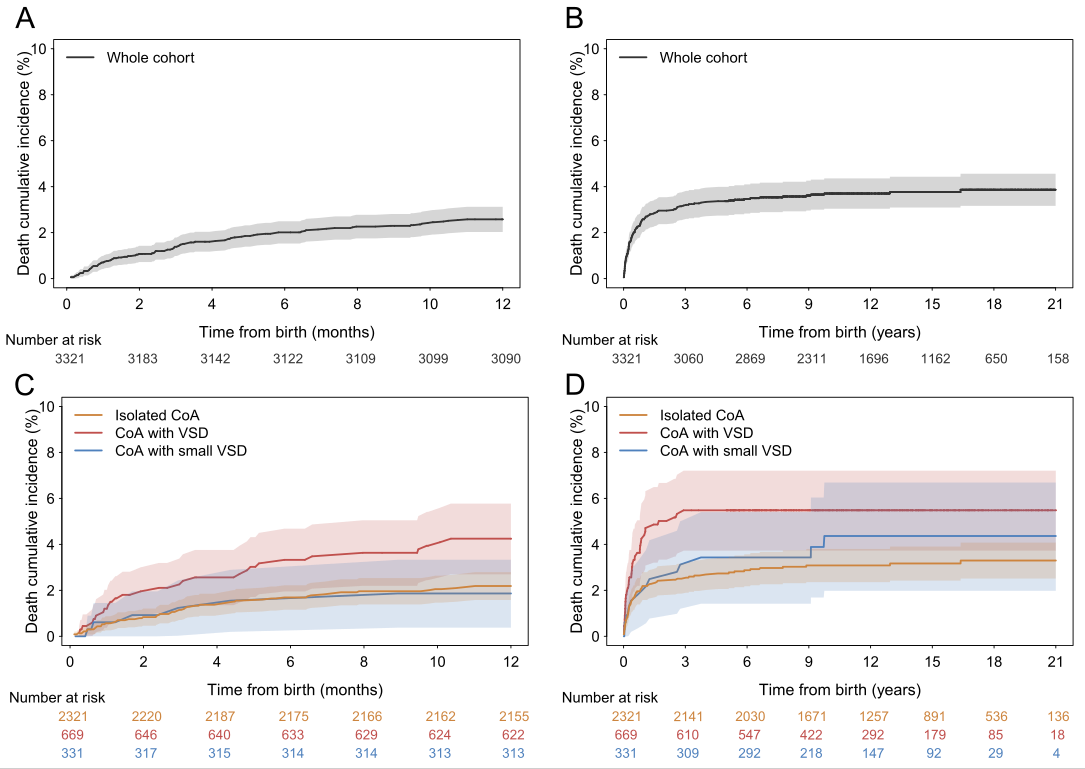


| **Supplementary Table 15.** Cumulative incidence of death up to maximum follow-up from birth | | |
| --- | --- | --- |
| **Time from birth (months)** | **Number at risk** | **Death cumulative incidence** |
| 0 | 3321 |  |
| 1 | 3214 | 0.7% (0.4%-1.0%) |
| 2 | 3183 | 1.1% (0.7%-1.4%) |
| 3 | 3158 | 1.4% (1.0%-1.8%) |
| 4 | 3142 | 1.6% (1.2%-2.0%) |
| 5 | 3131 | 1.9% (1.4%-2.3%) |
| 6 | 3122 | 2.0% (1.5%-2.5%) |
| 7 | 3114 | 2.1% (1.6%-2.6%) |
| 8 | 3109 | 2.3% (1.7%-2.8%) |
| 9 | 3107 | 2.3% (1.8%-2.8%) |
| 10 | 3099 | 2.4% (1.9%-2.9%) |
| 11 | 3094 | 2.6% (2.0%-3.1%) |
| 12 | 3090 | 2.6% (2.0%-3.1%) |
| **Time from birth (years)** | **Number at risk** | **Death cumulative incidence** |
| 0 | 3321 |  |
| 1 | 3090 | 2.6% (2.0%-3.1%) |
| 2 | 3072 | 3.0% (2.4%-3.5%) |
| 3 | 3060 | 3.2% (2.6%-3.8%) |
| 4 | 3048 | 3.3% (2.7%-4.0%) |
| 5 | 3036 | 3.4% (2.7%-4.0%) |
| 6 | 2869 | 3.5% (2.8%-4.1%) |
| 7 | 2697 | 3.5% (2.9%-4.2%) |
| 8 | 2489 | 3.6% (2.9%-4.2%) |
| 9 | 2311 | 3.6% (3.0%-4.3%) |
| 10 | 2096 | 3.7% (3.0%-4.4%) |
| 11 | 1892 | 3.7% (3.0%-4.4%) |
| 12 | 1696 | 3.7% (3.0%-4.4%) |
| 13 | 1520 | 3.8% (3.1%-4.4%) |
| 14 | 1348 | 3.8% (3.1%-4.4%) |
| 15 | 1162 | 3.8% (3.1%-4.4%) |
| 16 | 1006 | 3.8% (3.1%-4.4%) |
| 17 | 836 | 3.9% (3.2%-4.6%) |
| 18 | 650 | 3.9% (3.2%-4.6%) |
| 19 | 493 | 3.9% (3.2%-4.6%) |
| 20 | 324 | 3.9% (3.2%-4.6%) |
| 21 | 158 | 3.9% (3.2%-4.6%) |
| *For each time point, the table contains the number at risk at that time (did not die and were not lost due to data censoring) and the estimated mortality rate with 95% CI.* | | |

| **Supplementary Table 16.** Cumulative incidence of cardiac reintervention after coarctation of the aorta (CoA) repair, by type | | | | | | | | | | | | |
| --- | --- | --- | --- | --- | --- | --- | --- | --- | --- | --- | --- | --- |
|  | **Reintervention type** | | | | | | | | | | | |
|  | ***Any*** | | ***Surgical*** | | ***Catheter*** | | ***CoA repair redo*** | | ***PA arterioplasty or PA (de)banding*** | | ***Subaortic stenosis relief*** | |
|  | *N at risk* | *Cumulative incidence* | *N at risk* | *Cumulative incidence* | *N at risk* | *Cumulative incidence* | *N at risk* | *Cumulative incidence* | *N at risk* | *Cumulative incidence* | *N at risk* | *Cumulative incidence* |
| **Months from CoA repair** | | | | | | | | | | | | |
| 1 | 3014 | 2.7 (2.2-3.3) | 3023 | 2.4 (1.9-3) | 3084 | 0.3 (0.2-0.6) | 3048 | 1.6 (1.2-2.0) | 3068 | 0.8 (0.6-1.2) | 3093 | NA |
| 2 | 2920 | 4.7 (4-5.5) | 2962 | 3.3 (2.7-4) | 3013 | 1.6 (1.2-2) | 2963 | 3.3 (2.7-3.9) | 3030 | 1.0 (0.7-1.4) | 3059 | 0.0 (0.0-0.2) |
| 3 | 2818 | 7.0 (6.2-8) | 2909 | 4.0 (3.4-4.8) | 2927 | 3.2 (2.6-3.9) | 2866 | 5.4 (4.6-6.2) | 2989 | 1.3 (0.9-1.7) | 3022 | 0.1 (0.0-0.3) |
| 4 | 2757 | 8.4 (7.5-9.4) | 2878 | 4.4 (3.7-5.1) | 2870 | 4.4 (3.7-5.2) | 2807 | 6.6 (5.8-7.5) | 2962 | 1.4 (1.1-1.9) | 2998 | 0.1 (0.0-0.3) |
| 5 | 2682 | 9.9 (8.9-11) | 2834 | 4.9 (4.2-5.7) | 2800 | 5.7 (4.9-6.5) | 2735 | 8.0 (7.0-8.9) | 2924 | 1.6 (1.2-2.1) | 2965 | 0.1 (0.0-0.3) |
| 6 | 2630 | 11.2 (10.1-12.3) | 2803 | 5.3 (4.6-6.2) | 2750 | 6.7 (5.9-7.6) | 2685 | 9.0 (8.1-10.1) | 2903 | 1.7 (1.3-2.2) | 2942 | 0.2 (0.1-0.4) |
| 7 | 2588 | 11.8 (10.6-12.9) | 2765 | 5.7 (4.9-6.5) | 2713 | 7.1 (6.2-8.1) | 2645 | 9.6 (8.6-10.7) | 2876 | 1.7 (1.3-2.2) | 2914 | 0.2 (0.1-0.4) |
| 8 | 2555 | 12.4 (11.3-13.6) | 2740 | 6.1 (5.3-6.9) | 2687 | 7.5 (6.6-8.5) | 2615 | 10.1 (9.1-11.2) | 2857 | 1.8 (1.3-2.3) | 2894 | 0.3 (0.1-0.5) |
| 9 | 2519 | 13.0 (11.8-14.2) | 2709 | 6.4 (5.6-7.3) | 2657 | 7.8 (6.9-8.8) | 2582 | 10.5 (9.4-11.6) | 2836 | 1.8 (1.4-2.3) | 2871 | 0.3 (0.1-0.6) |
| 10 | 2491 | 13.3 (12.1-14.5) | 2679 | 6.6 (5.8-7.5) | 2630 | 8.0 (7-8.9) | 2554 | 10.7 (9.6-11.8) | 2807 | 1.9 (1.5-2.4) | 2843 | 0.3 (0.2-0.6) |
| 11 | 2471 | 13.6 (12.4-14.8) | 2661 | 6.7 (5.9-7.7) | 2614 | 8.1 (7.2-9.1) | 2538 | 10.9 (9.8-12.0) | 2788 | 2.0 (1.6-2.5) | 2827 | 0.4 (0.2-0.6) |
| 12 | 2445 | 14.0 (12.8-15.2) | 2642 | 6.9 (6-7.8) | 2590 | 8.4 (7.4-9.4) | 2514 | 11.2 (10.1-12.3) | 2770 | 2.1 (1.6-2.7) | 2810 | 0.4 (0.2-0.7) |
|  | ***Any*** | | ***Surgical*** | | ***Catheter*** | | ***CoA repair redo*** | | ***PA arterioplasty or PA (de)banding*** | | ***Subaortic stenosis relief*** | |
|  | *N at risk* | *Cumulative incidence* | *N at risk* | *Cumulative incidence* | *N at risk* | *Cumulative incidence* | *N at risk* | *Cumulative incidence* | *N at risk* | *Cumulative incidence* | *N at risk* | *Cumulative incidence* |
| **Years from CoA repair** | | | | | | | | | | | | |
| 1 | 2445 | 14.0 (12.8-15.2) | 2642 | 6.9 (6.0-7.8) | 2590 | 8.4 (7.4-9.4) | 2514 | 11.2 (10.1-12.3) | 2770 | 2.1 (1.6-2.7) | 2810 | 0.4 (0.2-0.7) |
| 2 | 2187 | 16.3 (15.1-17.7) | 2384 | 8.6 (7.7-9.7) | 2353 | 9.4 (8.3-10.4) | 2281 | 12.2 (11.1-13.4) | 2528 | 2.9 (2.3-3.5) | 2575 | 0.8 (0.5-1.2) |
| 3 | 1969 | 17.8 (16.4-19.2) | 2151 | 10.1 (9.1-11.3) | 2148 | 9.6 (8.6-10.7) | 2076 | 12.7 (11.5-13.9) | 2311 | 3.0 (2.5-3.7) | 2332 | 1.8 (1.3-2.3) |
| 4 | 1772 | 18.8 (17.4-20.2) | 1940 | 10.8 (9.7-11.9) | 1949 | 10.1 (9.1-11.3) | 1883 | 13.1 (11.9-14.4) | 2100 | 3.1 (2.5-3.8) | 2115 | 2.1 (1.6-2.7) |
| 5 | 1553 | 19.5 (18.1-21) | 1709 | 11.3 (10.1-12.5) | 1716 | 10.6 (9.5-11.7) | 1654 | 13.5 (12.3-14.8) | 1865 | 3.1 (2.5-3.8) | 1868 | 2.5 (1.9-3.2) |
| 6 | 1375 | 20.1 (18.6-21.6) | 1524 | 11.4 (10.2-12.6) | 1523 | 11.0 (9.9-12.2) | 1470 | 13.8 (12.6-15.1) | 1668 | 3.1 (2.5-3.8) | 1667 | 2.6 (2.0-3.3) |
| 7 | 1195 | 21.1 (19.6-22.6) | 1344 | 11.7 (10.6-12.9) | 1332 | 11.7 (10.6-13) | 1284 | 14.5 (13.2-15.9) | 1474 | 3.1 (2.5-3.8) | 1473 | 2.9 (2.3-3.6) |
| 8 | 1036 | 21.6 (20-23.1) | 1174 | 12.0 (10.8-13.2) | 1154 | 12.2 (10.9-13.4) | 1112 | 14.8 (13.5-16.2) | 1286 | 3.1 (2.5-3.8) | 1282 | 3.2 (2.6-4.0) |
| 9 | 886 | 22.1 (20.5-23.7) | 1010 | 12.4 (11.1-13.7) | 986 | 12.4 (11.1-13.7) | 956 | 15.0 (13.7-16.4) | 1105 | 3.1 (2.5-3.8) | 1101 | 3.5 (2.8-4.4) |
| 10 | 734 | 22.6 (21-24.3) | 851 | 12.7 (11.5-14.1) | 819 | 12.6 (11.3-13.9) | 792 | 15.3 (13.9-16.7) | 933 | 3.1 (2.5-3.8) | 930 | 3.6 (2.9-4.5) |
| 11 | 620 | 23.3 (21.6-25) | 720 | 13.1 (11.7-14.4) | 687 | 13.0 (11.7-14.4) | 667 | 15.7 (14.3-17.2) | 788 | 3.1 (2.5-3.8) | 786 | 3.9 (3.1-4.8) |
| 12 | 517 | 23.6 (21.8-25.3) | 595 | 13.4 (12.1-14.9) | 571 | 13.2 (11.8-14.6) | 551 | 16.0 (14.6-17.5) | 652 | 3.1 (2.5-3.8) | 651 | 4.0 (3.1-4.9) |
| 13 | 402 | 23.9 (22.1-25.7) | 462 | 13.6 (12.2-15.1) | 435 | 13.4 (12-14.9) | 423 | 16.2 (14.7-17.7) | 502 | 3.1 (2.5-3.8) | 497 | 4.0 (3.1-4.9) |
| 14 | 297 | 24.3 (22.4-26.1) | 340 | 13.6 (12.2-15.1) | 318 | 13.9 (12.4-15.4) | 310 | 16.6 (15.0-18.2) | 366 | 3.1 (2.5-3.8) | 365 | 4.0 (3.1-4.9) |
| 15 | 206 | 24.6 (22.6-26.5) | 233 | 13.6 (12.2-15.1) | 215 | 14.2 (12.6-15.9) | 214 | 16.9 (15.2-18.7) | 245 | 3.1 (2.5-3.8) | 246 | 4.0 (3.1-4.9) |
| 16 | 94 | 24.6 (22.6-26.6) | 111 | 14.1 (12.4-15.8) | 98 | 14.2 (12.6-15.9) | 98 | 16.9 (15.2-18.7) | 116 | 3.1 (2.5-3.8) | 118 | 4.0 (3.1-4.9) |
| *CoA, coarctation of the aorta; PA, pulmonary artery.*  *For each time point, the table contains the number left at risk at that time (did not have a reintervention, did not die and were not lost due to data censoring) and the estimated cumulative incidence functions of reintervention with 95% confidence interval.* | | | | | | | | | | | | |

| **Supplementary Table 17.** Days spent in hospital for coarctation of the aorta (CoA) patients during the first year of file and up to the 1ge of 18 years old. | | | | | | | | | |
| --- | --- | --- | --- | --- | --- | --- | --- | --- | --- |
|  | **Total time in hospital (days)** | | | | | | | | |
|  | ***Total (inpatient, outpatient and A&E)*** | | | ***Inpatient only*** | | | ***Outpatient only*** | | |
|  | **Patient number** | **Median (IQR)** | **Range** | **Patient number** | **Median (IQR)** | **Range** | **Patient number** | **Median (IQR)** | **Range** |
| **Monthly interval from birth** | | | | | | | | | |
| 1^st^ | 1766 | 13 (4-22) | (0-30) | 3084 | 11 (2-19) | (0-30) | 2524 | 0 (0-0) | (0-5) |
| 2^nd^ | 1773 | 2 (1-8) | (0-30) | 3066 | 0 (0-6) | (0-30) | 2488 | 0 (0-1) | (0-12) |
| 3^rd^ | 1782 | 1 (0-4) | (0-30) | 3058 | 0 (0-1) | (0-30) | 2489 | 0 (0-1) | (0-10) |
| 4^th^ | 1786 | 1 (0-2) | (0-30) | 3049 | 0 (0-0) | (0-30) | 2485 | 0 (0-1) | (0-11) |
| 5^th^ | 1793 | 1 (0-2) | (0-30) | 3042 | 0 (0-0) | (0-30) | 2490 | 0 (0-1) | (0-10) |
| 6^th^ | 1806 | 1 (0-2) | (0-30) | 3035 | 0 (0-0) | (0-30) | 2495 | 0 (0-1) | (0-10) |
| 7^th^ | 1812 | 0 (0-1) | (0-30) | 3031 | 0 (0-0) | (0-30) | 2506 | 0 (0-1) | (0-8) |
| 8^th^ | 1827 | 0 (0-1) | (0-30) | 3027 | 0 (0-0) | (0-30) | 2507 | 0 (0-1) | (0-8) |
| 9^th^ | 1833 | 0 (0-1) | (0-30) | 3024 | 0 (0-0) | (0-30) | 2513 | 0 (0-1) | (0-10) |
| 10^th^ | 1850 | 0 (0-1) | (0-30) | 3022 | 0 (0-0) | (0-30) | 2529 | 0 (0-1) | (0-11) |
| 11^th^ | 1852 | 0 (0-1) | (0-30) | 3019 | 0 (0-0) | (0-30) | 2536 | 0 (0-1) | (0-12) |
| 12^th^ | 1866 | 0 (0-1) | (0-30) | 3016 | 0 (0-0) | (0-30) | 2551 | 0 (0-1) | (0-8) |
|  | **Total time in hospital (days)** | | | | | | | | |
|  | ***Total (inpatient, outpatient and A&E)*** | | | ***Inpatient only*** | | | ***Outpatient only*** | | |
|  | **Patient number** | **Median (IQR)** | **Range** | **Patient number** | **Median (IQR)** | **Range** | **Patient number** | **Median (IQR)** | **Range** |
| **Yearly interval from birth** | | | | | | | | | |
| 1^st^ | 1766 | 26 (17-44) | (0-365) | 3084 | 16 (10-31) | (0-365) | 2524 | 5 (3-8) | (0-59) |
| 2^nd^ | 1887 | 4 (2-9) | (0-365) | 3030 | 0 (0-2) | (0-365) | 2559 | 3 (1-5) | (0-85) |
| 3^rd^ | 1892 | 3 (1-6) | (0-176) | 2870 | 0 (0-1) | (0-226) | 2575 | 2 (1-4) | (0-58) |
| 4^th^ | 1902 | 2 (1-5) | (0-205) | 2698 | 0 (0-0) | (0-205) | 2586 | 1 (1-4) | (0-64) |
| 5^th^ | 1876 | 2 (1-5) | (0-109) | 2499 | 0 (0-0) | (0-93) | 2396 | 1 (1-3) | (0-70) |
| 6^th^ | 1871 | 2 (1-4) | (0-69) | 2321 | 0 (0-0) | (0-62) | 2229 | 1 (1-3) | (0-33) |
| 7^th^ | 1819 | 2 (1-4) | (0-67) | 2101 | 0 (0-0) | (0-120) | 2017 | 1 (1-3) | (0-30) |
| 8^th^ | 1803 | 1 (1-3) | (0-315) | 1904 | 0 (0-0) | (0-315) | 1827 | 1 (0-3) | (0-43) |
| 9^th^ | 1625 | 1 (1-3) | (0-86) | 1713 | 0 (0-0) | (0-82) | 1646 | 1 (0-3) | (0-39) |
| 10^th^ | 1444 | 1 (0-3) | (0-85) | 1524 | 0 (0-0) | (0-84) | 1464 | 1 (0-2) | (0-25) |
| 11^th^ | 1283 | 1 (1-4) | (0-154) | 1355 | 0 (0-0) | (0-154) | 1300 | 1 (0-3) | (0-23) |
| 12^th^ | 1113 | 1 (1-3) | (0-96) | 1176 | 0 (0-0) | (0-71) | 1127 | 1 (0-2) | (0-48) |
| 13^th^ | 963 | 1 (1-3) | (0-71) | 1018 | 0 (0-0) | (0-70) | 975 | 1 (0-3) | (0-29) |
| 14^th^ | 815 | 1 (1-3) | (0-51) | 859 | 0 (0-0) | (0-48) | 827 | 1 (0-3) | (0-25) |
| 15^th^ | 645 | 1 (1-4) | (0-55) | 675 | 0 (0-0) | (0-47) | 656 | 1 (0-3) | (0-31) |
| 16^th^ | 491 | 1 (0-4) | (0-66) | 509 | 0 (0-0) | (0-54) | 501 | 1 (0-3) | (0-28) |
| 17^th^ | 329 | 1 (0-3) | (0-55) | 339 | 0 (0-0) | (0-37) | 335 | 1 (0-2) | (0-30) |
| 18^th^ | 171 | 1 (0-2) | (0-35) | 177 | 0 (0-0) | (0-34) | 173 | 1 (0-2) | (0-11) |
| *We required patients to be alive and have all data coverage (inpatient, outpatient and A&E), Inpatient data coverage and Outpatient data coverage for at least part of the year reported (row in table). Inpatient, outpatient and A&E data are available from 2000, 2003 and 2007 onwards in England patients, respectively. N=208 patients had no linkage to Inpatient data and were removed. N=18 patients who had Inpatient spell without a discharge age were removed from the inpatient associated hospital days. Of note, Accidents and Emergency (A&E) data was non-cardiac and only available from 2007. We did a sensitivity analysis and results revealed that including A&E or only led to a less than one-day difference in evaluating the total days spent in hospital.* | | | | | | | | | |

| **Supplementary Table 18.** Risk factors associated with cardiac reintervention after CoA repair, by type of reintervention from unadjusted analysis | | | | |
| --- | --- | --- | --- | --- |
| **Risk factors** | **Reintervention type** | | | |
|  | **Any (n=261)** | **CoA repair redo (n=172)** | **PA arterioplasty or PA (de) banding (n=37)** | **Subaortic stenosis relief (n=37)** |
| CoA repair pathway |  |  |  |  |
| Isolated CoA repair (surgical) | reference | reference | reference | reference |
| CoA repair by catheterization | 3.44 (2.2-5.32)*** | 4.38 (2.92-6.55)*** | 1.17 (0.22-6.33) | ^ |
| CoA repair with VSD closure | 1.31 (0.90-1.91) | 0.71 (0.35-1.41) | 1.19 (0.43-3.32) | 4.48 (1.93-10.36)*** |
| CoA repair with PA banding | 3.66 (2.45-5.78) | 1.53 (1.05-2.25)* | 24.65 (12.83-47.38)*** | 1.63 (0.42-6.37) |
| Additional cardiac risk factor | 1.69 (1.07-2.67)*** |  |  |  |
| Preterm birth |  | 2.11 (1.31-3.39)** |  |  |
| Congenital non-cardiac comorbidity |  |  | 1.85 (1.22-2.80)** |  |
| Low weight at first procedure | 1.67 (1.17-2.38)** | 1.86 (1.18-2.92)** | 2.46 (1.28-4.73)** |  |
| SoI marker at first procedure | 1.69 (1.22-2.35)** | 1.48 (1.07-2.02)* | 2.56 (1.73-3.76)*** |  |
| Age at first cardiac procedure (days) |  |  | 0.98 (0.97-0.99)** | 0.98 (0.97-0.99)*** |
| *Significance level (p-value): 0.05 * 0.01 ** 0.001 *** ^ N/A due to no occurrence*  *n=1,563 patient included (born from 2009 onwards undergoing CoA repair). In addition to the CoA repair subtypes, we only listed the risk factor which is significant in the univariable model (p<0.05).*  *CoA, coarctation of the aorta; PA, pulmonary artery; SoI, severity of illness; VSD, ventricular septal defect.* | | | | |
